# Design, Synthesis and Characterization of Covalent KDM5 Inhibitors

**DOI:** 10.1002/anie.201810179

**Published:** 2018-12-07

**Authors:** Saleta Vazquez‐Rodriguez, Miranda Wright, Catherine M. Rogers, Adam P. Cribbs, Srikannathasan Velupillai, Martin Philpott, Henry Lee, James E. Dunford, Kilian V. M. Huber, Matthew B. Robers, James D. Vasta, Marie‐Laetitia Thezenas, Sarah Bonham, Benedikt Kessler, James Bennett, Oleg Fedorov, Florence Raynaud, Adam Donovan, Julian Blagg, Vassilios Bavetsias, Udo Oppermann, Chas Bountra, Akane Kawamura, Paul E. Brennan

**Affiliations:** ^1^ Structural Genomics Consortium & Target Discovery Institute University of Oxford NDM Research Building Roosevelt Drive Oxford OX3 7DQ and OX3 7FZ UK; ^2^ Botnar Research Center Nuffield Department of Orthopedics Rheumatology and Musculoskeletal Sciences NIHR Oxford BRC University of Oxford Oxford OX3 7DQ UK; ^3^ FRIAS—Freiburg Institute of Advanced Studies 79104 Freiburg Germany; ^4^ Chemistry Research Laboratory University of Oxford 12 Mansfield Road Oxford OX1 3TA UK; ^5^ Promega Corporation 2800 Woods Hollow Road Fitchburg WI 53711 USA; ^6^ Target Discovery Institute Nuffield Department of Medicine University of Oxford Roosevelt Drive OX3 7FZ Oxford UK; ^7^ Cancer Research (UK) Cancer Therapeutics Unit The Institute of Cancer Research 15 Cotswold Road London SM2 5NG UK

**Keywords:** covalent inhibitors, epigenetics, KDM5, lysine demethylase

## Abstract

Histone lysine demethylases (KDMs) are involved in the dynamic regulation of gene expression and they play a critical role in several biological processes. Achieving selectivity over the different KDMs has been a major challenge for KDM inhibitor development. Here we report potent and selective KDM5 covalent inhibitors designed to target cysteine residues only present in the KDM5 sub‐family. The covalent binding to the targeted proteins was confirmed by MS and time‐dependent inhibition. Additional competition assays show that compounds were non 2‐OG competitive. Target engagement and ChIP‐seq analysis showed that the compounds inhibited the KDM5 members in cells at nano‐ to micromolar levels and induce a global increase of the H3K4me3 mark at transcriptional start sites.

Histone proteins can be covalently modified by a plethora of post‐translational marks including methylation. These modifications modulate chromatin structure which impacts gene expression, chromosome packaging and DNA damage repair. Methylation is a reversible process regulated by histone methyltransferases (HMTs) and lysine demethylases (KDMs) that control the methylation state (mono‐, di‐, or tri‐methylation) of lysine residues in histone tails.^1^ The larger class of KDMs, the Jumonji‐C domain containing lysine demethylases (JmjC‐KDMs) utilize Fe^II^ and 2‐oxoglutarate (2‐OG) to oxidise methylated lysine substrates to form an unstable hemiaminal which fragments to the demethylated lysine and formaldehyde.^2^

JmjC‐KDMs are a family of 20 human enzymes which are further categorized into subfamilies KDM2‐7.^3^ There are four enzymes in the KDM5 subfamily (KDM5A‐D) which can all demethylate di‐ and tri‐methylated lysine 4 of histone 3 (H3K4me2/3), ubiquitous histone marks necessary for transcriptional activation. Various studies have linked the KDM5s to different cancers, in particular overexpression of KDM5B has been observed in prostate, gastric, breast, ovarian and hepatic cancer cells.^4^ KDM5B also has a role in development by blocking differentiation of embryonic and hematopoietic stem cells.^5^ The KDM5s have therefore become increasingly popular targets for chemical probe and drug discovery to gain a greater understanding of their role in biology and as oncology targets.

Several small‐molecule inhibitors of KDM5s have been reported.^6^ They bind to KDM5s through coordination to the active site Fe^II^ in the 2‐OG binding site and form a salt bridge with a conserved lysine or hydrogen bond to a conserved tyrosine residue in the KDM5 subfamily. Although nanomolar enzyme IC_50_ values are reported, cellular activities are commonly much weaker which has been attributed to competition of the inhibitors with higher levels of competing substrate in cells (2‐OG≈1 mm)^7^ compared to that used in biochemical assay conditions (low μm range), a factor known to reduce kinase inhibitor efficacy.^8^ Although KDM5 inhibitors have shown good selectivity over KDM2/3/6, selectivity over the KDM4 subfamily has proven more challenging as the primary binding residues of the catalytic domain are conserved in KDM4 and KDM5. Selectivity has only been achieved through structural changes in the secondary shell. For example, cyanopyrazole **CPI‐455** is a potent, selective KDM5 inhibitor with nanomolar enzyme IC_50_, but micromolar cellular IC_50_.^9^ Further optimisation led to *tert*‐butyl pyrazole **1** 10 which showed improved cellular activity (Figure 1 B). Potent KDM5 inhibitors, such as **2**, incorporating a 8‐pyrazolopyridopyrimidinone core were published, however this series suffered from poor selectivity over KDM4 and poor cellular potency despite cellular permeability.^11^

**Figure 1:**
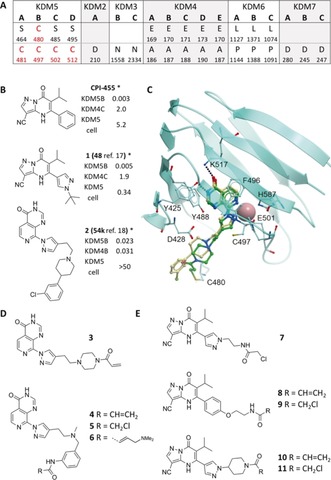
A) KDM alignment at positions C480 and C497 (KDM5B numbering). B) Reported KDM5 inhibitors **CPI‐455**, **1** and **2**. C) Overlay of compound **2** docked in the X‐ray structure of **3** (green) in KDM5B (PDB ID 6EIN). D) PP compounds. E) PZ compounds. *IC_50_ (μm).

Here we report two series of KDM5 selective covalent inhibitors based on cyanopyrazole **CPI‐455** and pyridopyrimidinone **2** with improved selectivity that target key cysteines exclusive to the KDM5s. Irreversible binding of the protein to the inhibitor resulted in reduced competition with the 2‐OG cosubstrate and maintained their cellular activity despite low cellular permeability. Covalent inhibitors are potentially more selective and potent, with better cellular efficiency and can require less frequent and lower doses, although this can be offset by poor selectivity with the cellular cysteinome. Covalent inhibitors can also be used as proteomic tools for both covalent and non‐covalent drugs.^12^

Based on alignments of the JmjC‐KDMs, two cysteine residues were identified as potential nucleophiles for covalent modifications in the KDM5 family. C497 in KDM5B was identified as a non‐catalytic residue close to the binding site present only in the KDM5 members. In addition, C480 was identified as a unique residue in KDM5B, so improved selectivity could be achieved over KDM4s as well as KDM5A/C/D (Figure 1 A). The irreversible binding of the covalent inhibitors to KDM5s could also reduce competition with 2‐OG to improve their cellular activity.

C480 is positioned close to the binding site of previously reported inhibitor^11^ **2** and was suitable for the design of cysteine‐selective electrophiles based on this scaffold. 8‐Pyridopyrimidinone‐based covalent inhibitors **3**–**6** (PP series) (Figure 1 D) were synthesised (Schemes S1, S2 in the Supporting Information) and acrylamide **3** was co‐crystallised with KDM5B to determine the binding mode. Bidentate metal coordination and salt bridging/hydrogen bonding to K517/Y425 was observed with inhibitor **3** and a covalent bond was seen between the C480 sulfur and acrylamide β‐carbon (Figure 1 C).

A second series of KDM5 covalent inhibitors **7**–**11** (PZ series) (Figure 1 E) were also designed and synthesized (Schemes S3, S4) to target C497. X‐ray crystal structures of **7** and **10** showed the reactive chloroacetyl and acrylamide moieties within proximity of C497 sulfur (**7**, 5.8 Å; **10**, 11.7 Å) (Figure S1) although no covalent bond was seen in the crystal structure. The acrylamide of **10** was positioned further from C497 however compound flexibility could position it within covalent bonding distance. Both compounds maintained the key coordination of the nitrile to the metal centre and H‐bonding interactions of the carbonyl oxygen.

Since covalent inhibitors possess time‐dependent inhibition due to the kinetics of covalent binding to the protein, their activity was better assessed through determination of the kinetic parameter *k*_inact_/*K*_i_, rather than a simple IC_50_. These parameters were calculated by using an established method to derive *K*_i_ and *k*_inact_ directly from time‐dependant IC_50_ values (Table S1). IC_50_ values were measured using an AlphaScreen assay^13^ by pre‐incubating the compounds with KDM5B at different time points ranging from 0 to 120 minutes and the *k*_inact_ and *K*_i_ values determined as previously described.^14^

In the PP series, chloroacetamide **5** was the most potent with the highest *k*_inact_/*K*_i_ (40×10^3^ m ^−1^ s^−1^) and although acrylamide **4** was less active than **5** with (*k*_inact_/*K*_i_ 7.4×10^3^ m ^−1^ s^−1^) it showed a drop in IC_50_ of 12‐fold after an hour incubation (Figure 2 A). Dimethylamino crotonamide **6** was less potent than the acrylamide **4** with a *k*_inact_/*K*_i_ of 6.1×10^3^ m ^−1^ s^−1^. Acrylamide **4** was 5‐fold more potent than the corresponding piperazine acrylamide **3** (*k*_inact_/*K*_i_ 1.4×10^3^ m ^−1^ s^−1^).

**Figure 2:**
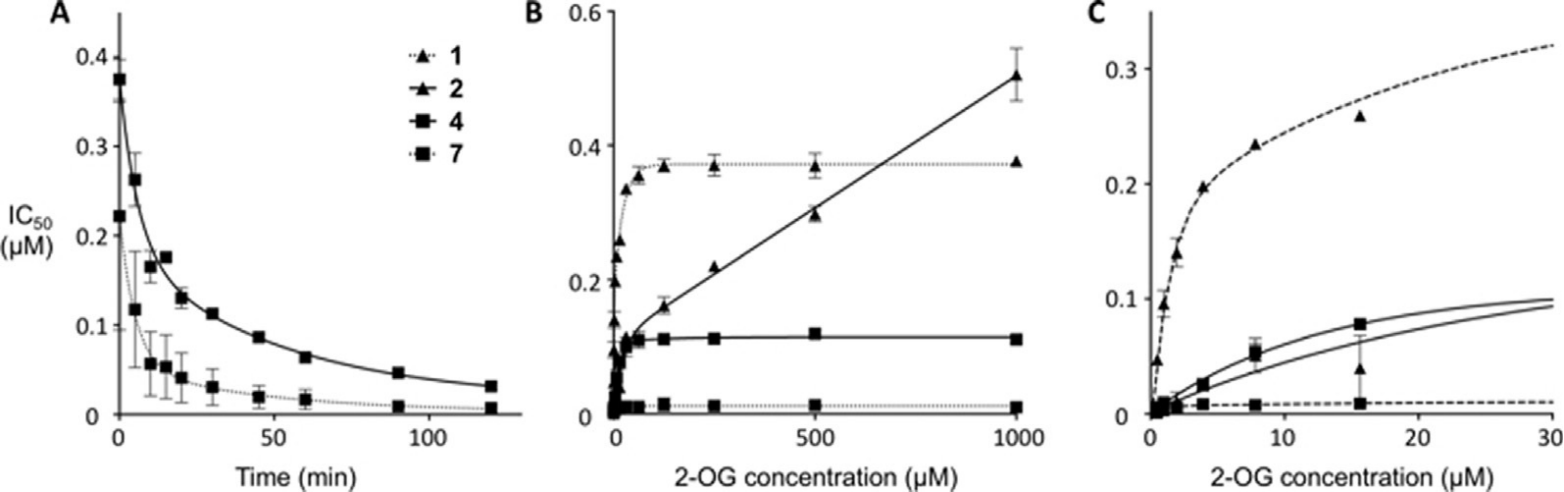
A) Time‐dependent inhibition of covalent inhibitors **4** and **7**. B,C) 2‐OG competition assay with non‐covalent variants (**1** and **2**) and covalent inhibitors (**4** and **7**).

In the PZ series, chloroacetamide **7** showed the greatest *k*_inact_/*K*_i_ (25×10^3^ m ^−1^ s^−1^) with an IC_50_ drop of 27‐fold upon pre‐incubation (Figure 2 A). Both acrylamide **10** and chloroacetamide **11** had comparable *k*_inact_/*K*_i_ values of 20×10^3^ and 19×10^3^ m ^−1^ s^−1^, while the phenyl analogues **8** and **9** showed the lowest *k*_inact_/*K*_i_ ratio with 6.9×10^3^ and 5.8×10^3^ m ^−1^ s^−1^, respectively. The larger drop in IC_50_ with the PZs compared to the PPs was likely due to the greater contribution of covalency to their potency. Overall, compounds in both series possessed time‐dependent inhibition through covalent binding to KDM5B leading to potent nanomolar IC_50_ values.

As well as improving their potency, the addition of a covalent electrophile to the inhibitors was expected to reduce competition with 2‐OG. IC_50_ values of both non‐covalent (**1** and **2**) and covalent inhibitors (**4** and **7**) were therefore determined at different concentrations of 2‐OG up to 1000 μm (Figure 2 B,C).

In the PP series, the non‐covalent analogue **2** showed an approximately 500‐fold drop in potency from 0.5 μm up to 1000 μm while the covalent inhibitor **4** showed only an 18‐fold lower potency at the high 2‐OG concentration. At 1 mm 2‐OG, **4** was 4‐fold more potent with an IC_50_ of 110 nm than **2** which we attribute to improved competition from 2‐OG compared to **2** by a factor of 25.

As reported,^9^ even the non‐covalent cyanopyrazoles **CPI‐455** and **1** are less competed by 2‐OG than the PP series, with compound **1** showing a drop in potency of only 8‐fold in the range of 2‐OG concentrations measured. As PZ compound **1** and PP compound **2** have similar binding modes based on their crystal structures, it is not clear why they show such different competition behaviour. But the covalent PZ inhibitor **7** was significantly more potent than non‐covalent PZ **1** with an IC_50_ at the top 2‐OG concentration of 10 nm and showed only a small shift in potency of 4‐fold with 2‐OG concentration. The addition of covalent inhibition to the PZs improved the potency as well as the competition with 2‐OG. Covalent inhibitors therefore represent an excellent strategy for solving the issues with KDM inhibitors in which high 2‐OG competition could explain their poor cellular activities.

A panel of JmjC KDMs was tested by AlphaScreen to determine the selectivity of the compounds (Table S2). IC_50_ values were measured at 120 minutes when the inhibition of the covalent inhibitors against KDM5B had levelled off (Figure 2 A). All the compounds from both series presented selectivity towards KDM5s, with the highest potency for the most closely related KDM5A and 5B. For the PZ series, the most potent compounds against KDM5B contained the 2‐chloroacetamide group, with **7** being the most potent of the PZ series (KDM5A/B IC_50_ 10 nm) and showed the best selectivity profile (Table S3). In the PP series the covalent compounds tested showed high activity with IC_50_ in the low nanomolar range against most KDM5 family members.

Both PP and PZ series showed the greatest selectivity over KDM2A and 3A with selectivity from 200‐ to 1500‐fold. The covalent inhibitors also demonstrated good selectivity over the most closely related KDM4 subfamily members. In the PP series, methylbenzylamine covalent inhibitors **4**–**6** showed greater than 20‐fold selectivity over the KDM4 family, in particular **5** was more than 60‐fold less active on KDM4B. We attribute this selectivity for KDM5B with the covalent PP inhibitors to binding to C480. In the PZ series, all compounds were more than 50‐fold selective over all the KDM4 family members, with compounds **7** and **10** having >500‐fold selectivity over KDM4A/B.

When comparing the activity of the compounds in the KDM5 family members, both series were equipotent against KDM5A/B/D, but 30–100‐fold selective over KDM5C. In the PP series, compound **6** was the most selective with greater than 7‐fold selectivity for KDM5B over KDM5A/C/D which could be explained by specific binding to C480. In the PZ series, the most selective compound was chloroacetamide **11** showing the highest selectivity for KDM5B with more than 100‐fold over KDM5C.

Covalent binding of the inhibitors with KDM5B was confirmed through MS‐labelling experiments (Figure 3). PP compound **3** was incubated with KDM5B and the resulting MS spectra showed a minor set of peaks relating to unmodified KDM5B (*m/z* 55 153) and a major peak relating to protein–inhibitor adduct with the correct mass shift (Δ*m/z* 379) (Figure 3 A). Compounds **4**–**6** were incubated with KDM4B and KDM5C under the same conditions as with KDM5B (Figure S2) and MS showed only unmodified protein peaks as expected for these proteins that do not contain a cysteine at C480 as in KDM5B. The specific covalent binding to KDM5B explains the improved selectivity over KDM5C and KDM4B compared to non‐covalent inhibitor **2**.

**Figure 3:**
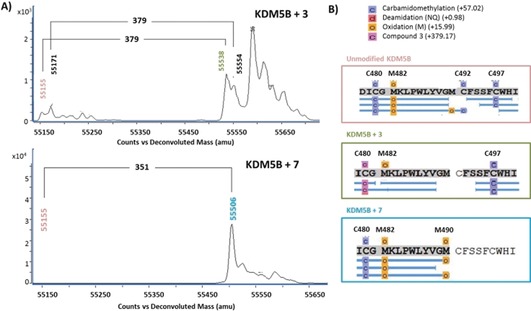
A) Intact mass spectra of covalent binding by MS. B) Peptide mapping of KDM5B after treatment with compounds **3** and **7**. Observed peptides for native protein (light red box), compound **3** adduct (green box) and compound **7** adduct (light blue box).

Digestion of the protein–inhibitor adduct and further analysis by LCMS/MS allowed mapping of the inhibitor reactive residue, confirming that compound **3** was binding specifically to the desired C480 and not to other cysteines in KDM5B (Figure 3 B, green box) in agreement with X‐ray crystal structure (Figure 1 C).

Similarly, incubation of **7** with KDM5B resulted in protein MS peaks from addition of the compound (Δ*m/z* 351) (Figure 3 A). LC‐MS/MS analysis (Figure 3 B) showed that although there was good coverage of the native protein (>90 %) including 3 peptides containing C497, with the inhibitor modified KDM5B, the peptide containing the target C497 was the only sequence that was not observed (Figure 3 B light blue box). As this peptide was observed in the unmodified protein, the addition of the compound appears to have prevented the detection of the peptide in the covalent adduct suggesting that C497 was the site of binding. Compounds **4**, **7** and **9** were also incubated with PCAF^15^ and NUDT7 (PDB ID 5QHH), two enzymes known to have covalent inhibitors, but no labelling was observed, demonstrating the selectivity for KDM5B (Figure S3).

With confirmation of potent and selective binding to recombinant KDM5 targets, we wanted to show cellular target engagement. We developed a PZ‐based cell‐permeable fluorescent tracer assay to quantify the engagement of compounds with KDM targets that are fused to NanoLuciferase.^16^ An aliphatic amino‐terminal linker on a PZ derivative was attached to the fluorescent dye NanoBRET_590_® to generate a tracer compound that interacts with the NanoLuc‐fused KDMs (Figure S4).

The tracer was tested in transfected NanoLuc‐KDM4s, KDM5s and KDM6B in the presence of compound **1**, showing that the tracer produced significant BRET signal in a dose‐dependent manner with all the KDM5 family members (KDM5A‐D), but no BRET signal for any of the KDM4s or KDM6B, demonstrating the selectivity of the tracer (Figure S5). Cellular activity of the most relevant PP and PZ compounds was then evaluated (Table 1, Figure 4 A, Figure S6). In the PZ series, phenyl‐containing compounds **8** and **9**, showed the best activity in cells (0.53 and 0.30 μm). However, compounds **7**, **10** and **11** showed micromolar IC_50_ values, which are more than a 100‐fold weaker than their in vitro activity, probably due to their low logP and poor cell permeability.

**Figure 4:**
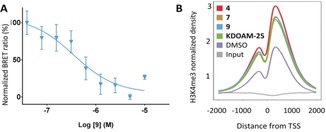
A) Cellular activity of compound **9** by NanoBRET assay. B) Distribution of H3K4me3 around transcriptional start sites (TSS). Densities of ChIP‐seq reads for H3K4me3 and input in HEK293 cells treated with DMSO and compounds **4**, **7**, **9** and non‐covalent reference compound KDOAM‐25.

**Table 1:** Biochemical and cellular properties of the PZ and PP series.

Cmpd	*c*log*P* ^[a]^	*P*_app_ ^[b]^	IC_50_ (2‐OG 1 μm) ^[d]^	IC_50_ (2‐OG 1 mm) ^[d]^	2‐OG shift^[e]^	KDM5B nano‐ BRET IC_50_ [μm]	ChIP‐seq conc [μm]
**2**	1.1	11.8	0.003	0.506	169	8.8	*ND*
**3**	−1.8	<1.5	0.184	*ND*	*ND*	>30	*ND*
**4**	−1.5	<1.5	0.009	*ND*	*ND*	10.6	*ND*
**5**	−0.94	<1.5	0.01	0.11	11	>30	2.0
**6**	−0.58	<1.5	0.004	0.094	26	>30	*ND*
							
**CPI‐455**	1.3	17.2^[c]^	0.003^[c]^	*ND*	2.0^[c]^	0.40	*ND*
**1**	0.65	7.5^[c]^	0.015	0.377	3.9	0.34	*ND*
**7**	−0.74	<0.76	0.007	0.010	2.0	2.5	2.0
**8**	0.68	<0.76	0.049	0.041	1.8	0.53	2.0
**9**	0.92	<0.76	0.065	0.059	1.9	0.30	*ND*
**10**	−0.89	<0.76	0.017	0.017	2.4	5.5	*ND*
**11**	−1.3	<1.4	0.009	0.008	1.3	8.6	*ND*

[a] Calculated with ChemDraw version 16.0.1.4. [b] Determined in Caco‐2 cells. Values are A to B (10^−6^ cm s^−1^). [c] Previously reported values in MDCK cells.^9^, ^10^ [d] AlphaScreen in vitro activity in KDM5B. [e] Calculated as ratio of IC_50_ values determined at 2‐OG concentrations of 1 mm and 1 μm.

Compound **4** was the only example of the PP series that showed some activity in cells, with an IC_50_ of 10.6 μm, in the same range as the non‐covalent compound **2** (IC_50_ 8.8 μm). For both the PP and PZ compounds, the similar cellular potency compared to their non‐covalent analogues can be explained due to their high polarity as measured clogP and poor cell permeability as measured by Caco‐2 (Table 1). Non‐covalent compounds **CPI‐455**, **2** and **3** have good permeability (Caco‐2 AB 7.5–17.2×10^−6^ cm s^−1^) but despite being much less cell permeable (Caco‐2 AB<1.5×10^−6^ cm s^−1^), the covalent compounds are as potent in cells as their non‐covalent analogues, presumably due to their better 2‐OG competition.

Immunofluorescence‐based assays have been previously used to analyse global changes in H3K4me3 as a measure of KDM5 activity, but the global change can be affected by other factors such as cytotoxicity.^17^ We therefore employed chromatin immunoprecipitation and sequencing (ChIP‐seq) as a more accurate method to quantify the H3K4me3 level at transcriptional start sites (TSS) as a read‐out for inhibition of KDM5 activity. Cytotoxicity of the compounds was first assessed to show that with the exception of **5**, compounds were not cytotoxic in HEK cells (Figure S7). Compounds **4**, **7** and **9** were selected for H3K4me3 ChIP‐seq experiments and quantification.^18^

After sequencing and normalisation, read coverage was evaluated and coverage plots were plotted across all genes revealing significant increases of H3K4me3 levels around TSS (Figure 4 B). ARID3B, known to be highly expressed in HEK cells,^19^ was chosen as a representative gene and all three compounds doubled H3K4me3 levels (Figure S8).

In conclusion, covalent inhibitors in the PP and PZ series that target distinctive cysteine residues in the KDM5 family has been shown to target lysine demethylases to overcome high cellular 2‐OG levels and improve KDM selectivity. Target engagement in cells was demonstrated using a novel NanoBRET assay and functional effects of the covalent inhibitors was shown by increase in H3K4me3 at TSS in HEK293 cells.

## Conflict of interest

The authors declare no conflict of interest.

## Supporting information

As a service to our authors and readers, this journal provides supporting information supplied by the authors. Such materials are peer reviewed and may be re‐organized for online delivery, but are not copy‐edited or typeset. Technical support issues arising from supporting information (other than missing files) should be addressed to the authors.

Supplementary
