# Supplementary material for: Design, Synthesis and Characterization of Covalent KDM5 Inhibitors
Source: Angew Chem Int Ed Engl. 2018 Dec 7;58(2):515–9. doi: 10.1002/anie.201810179 (PMC6391970; doi:10.1002/anie.201810179)
Supplement: Supplementary file 1 — Supplementary [file ANIE-58-515-s001.pdf]

Angewandte Chemie
Eine Zeitschrift der Gesellschaft Deutscher Chemiker

Supporting Information

Design, Synthesis and Characterization of Covalent KDM5 Inhibitors

Saleta Vazquez-Rodriguez[+], Miranda Wright[+], Catherine M. Rogers, Adam P. Cribbs, Srikannathasan Velupillai, Martin Philpott, Henry Lee, James E. Dunford, Kilian V. M. Huber, Matthew B. Robers, James D. Vasta, Marie-Laetitia Thezenas, Sarah Bonham, Benedikt Kessler, James Bennett, Oleg Fedorov, Florence Raynaud, Adam Donovan, Julian Blagg, Vassilios Bavetsias, Udo Oppermann, Chas Bountra, Akane Kawamura, and Paul E. Brennan**

anie_201810179_sm_miscellaneous_information.pdf

Table of Contents

General Experimental ... 2

Solvents and reagents ... 2

Purification and chromatography ... 2

Characterisation ... 2

General synthetic procedures: Synthesis of PP series ... 3

Synthesis of piperazine acrylamide 3 ... 3

Scheme S1. Synthesis of piperazine acrylamide 3 ... 3

Synthesis of methyl benzylamine inhibitors 4-6 ... 3

Scheme S2. Synthesis of methyl benzylamines 4-6 ... 4

General synthetic procedures: Synthesis of PZ series ... 8

Scheme S3 Synthetic procedure for the preparation of intermediate 28 ... 8

Scheme S4 Synthesis of PZ covalent inhibitors 7-11. ... 9

General procedure I: Synthesis of modified boronic esters (29 and 30) ... 9

General procedure II: Suzuki Coupling (31, 32 and 35). ... 9

General procedure III: Boc deprotections (33, 34 and 36) ... 10

General procedure IV: Final acylation step (7-11) ... 10

Example of synthesis of modified boronic ester. ... 10

Example of Suzuki coupling ... 11

Example of Boc-deprotection ... 12

Example of acylation ... 12

Biological and Biochemical methods ... 14

Protein purification ... 14

Protein Crystallisation, X-ray Data Collection and Structure Determination ... 14

KDM alpha screen selectivity ... 15

Timecourse KDM5B inhibition and Kinact/Ki calculations ... 15

KDM5B 2-OG competition assay ... 15

Covalent binding RapidFire ... 16

Mapping ... 16

NanoLuciferase Bioluminescent Resonance Energy Transfer (NanoBRET™) Assay ... 16

Cytotoxicity assessment ... 16

ChIP-seq ... 16

Data Processing of ChIP-Seq ... 17

Statistical analysis of data. ... 17

Supplementary Figures and Tables ... 18

Table S1 k_{inact} and K_i values for compounds 3-11 ... 18

Table S2 *In vitro* selectivity of the PZ and PP series ... 18

Table S3. Selectivity Index (SI) of Inhibitors over KDM5B ... 18

Figure S1. Co-crystal structures of compounds 7 and 10 in KDM5A ... 19

Figure S2. Mass Spectra of compound 6 incubated with KDM5B, KDM4B and KDM5C ... 19

Figure S3. Mass Spectra of compounds incubated with enzymes with known reported covalent inhibitors. ... 20

Figure S4. Titration of NanoBRET tracer with KDM5B-NanoLuc ... 20

Figure S5. Selectivity of KDM5 NanoBRET tracer 21
Figure S6. Cellular IC50 in the NanoBRET assay in HEK293 cells for PP and PZ compounds 21
Figure S7. Cytotoxicity of PZ and PP in HEK293 cells 22
Figure S8 Genomic snapshot of ARID3B 22
Table S4. AlphaScreen assay conditions for the KDM selectivity panel 23
Table S5. Crystallographic statistics for Compounds 3, 7 and 10 23
Figure S9. Electron density of compounds 3, 7 and 10 in co-crystal structures with KDM5B 24
References 24
Complete References from Manuscript 24

Chemical Synthesis

General Experimental

Solvents and reagents

All solvents were purchased from commercial sources and used without purification (HPLC or analytical grade). Anhydrous solvents were purchased from Acros Organics and stored under a nitrogen atmosphere with activated molecular sieves. NanoBRET 590 SE was obtained from Promega Corp. Madison. Standard vacuum line techniques were used and when anhydric conditions were needed, glassware was flame dried prior to use. Deionised water was sourced using an Elga DV 25 system. Organic solvents were dried during workup using anhydrous Na_2SO_4 or Phase separator cartridges.

Purification and chromatography

Thin Layer Chromatography (TLC) was performed on Merck Kieselgel 60 F254 0.25 mm precoated aluminium plates. Plates were visualised using UV light (λ = 254 nm or 365 nm) and/or staining with Ninhydrin (1 M, EtOH) or 1% aq. KMnO4. Normal-phase silica gel chromatography was carried out using Biotage Isolera One flash column chromatography system (LPLC) using prepacked SNAP-Ultra, amino-functionalised or KP-Sil silica columns. Reverse-phase high pressure liquid chromatography (RP-HPLC) was performed using a Waters system equipped with a Waters 2545 Binary Gradient Module, a SecurityGuard™ ULTRA cartridges for EVO-C18 UHPLC HPLC, Kinetex 5 µM EVO C18 100 Å 100 x 3.0 mm column and a Waters SQ Detector 2 using the stated eluent system.

Characterisation

NMR spectra were recorded using a Bruker Avance 400 MHz spectrometer in the deuterated solvent stated. Chemical shifts (δ) are quoted in parts per million (ppm) and using the residual non-deuterated solvent signal as an internal reference. Multiplicities are denoted as singlet (s), doublet (d), triplet (t), quartet (q), pentaplet (p), and derivatives thereof and multiplets as (m). The abbreviation br denotes a broad resonance peak. Coupling constants recorded as Hz and round to the nearest 0.1 Hz. Low Resolution mass spectra were recorded on a Waters SQ Detector 2 (LC-MS) and purity (≥ 95 %) determined by reverse-phase high pressure liquid chromatography (RP-HPLC) using a Kinetex 5 mm EVO C18 100 A˚ LC Column 30 3 2.1 mm column or a Phenomenex Synergi 2.5 mm Max-RP 100 A˚ L. High Resolution Mass Spectrometry (HRMS) was recorded using an Agilent 6530 QTOF. Chromolite SemiPrep RP18-e 10x100 mm and Phenomenex 5µ EVO C18 100A 21.2x150 mm columns were used for the preparative HPLC purifications using a gradient program; Eluent I: acetonitrile/water = 5/95 with 20 mM ammonium acetate buffer, pH 6.0, Eluent II: acetonitrile/water = 80/20 with 20 mM ammonium acetate buffer, pH 6.0. LCMS measurements were carried out using a WATERS sunfire C18 column using electrospray ionization, operating in the positive ion mode; separation was achieved using a linear gradient of solvent A (water + 0.01% CF3CO2H) and solvent B (acetonitrile + 0.01%

CF3CO2H), eluting at a flow rate of 1 mL/min and monitoring at 254 nm: 0% B over 2 min, 0% B to 100% B over 16 min and 100% B over 2 min.

Compound names were generated using ChemBioDraw Ultra v15 systematic naming.

General synthetic procedures: Synthesis of PP series

Synthesis of piperazine acrylamide 3

Piperazine acrylamide **3** was synthesised according to Scheme 1 through intermediate **19** which was described in the literature.[1]

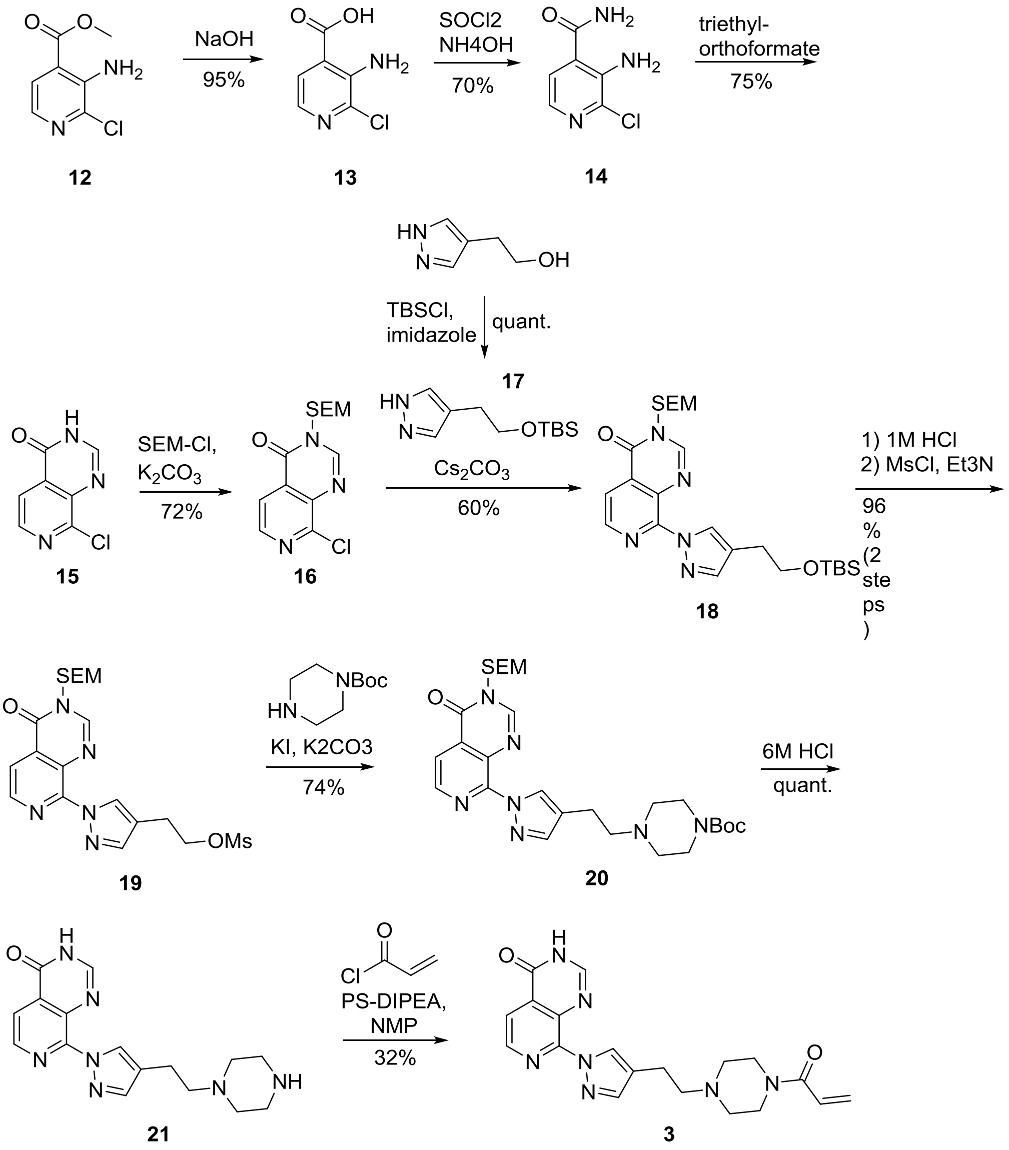

Scheme S1. Synthesis of piperazine acrylamide 3

Synthesis of methyl benzylamine inhibitors 4-6

Methyl benzylamines **4-6** were synthesised according to Scheme 2 from the known intermediate **19**.

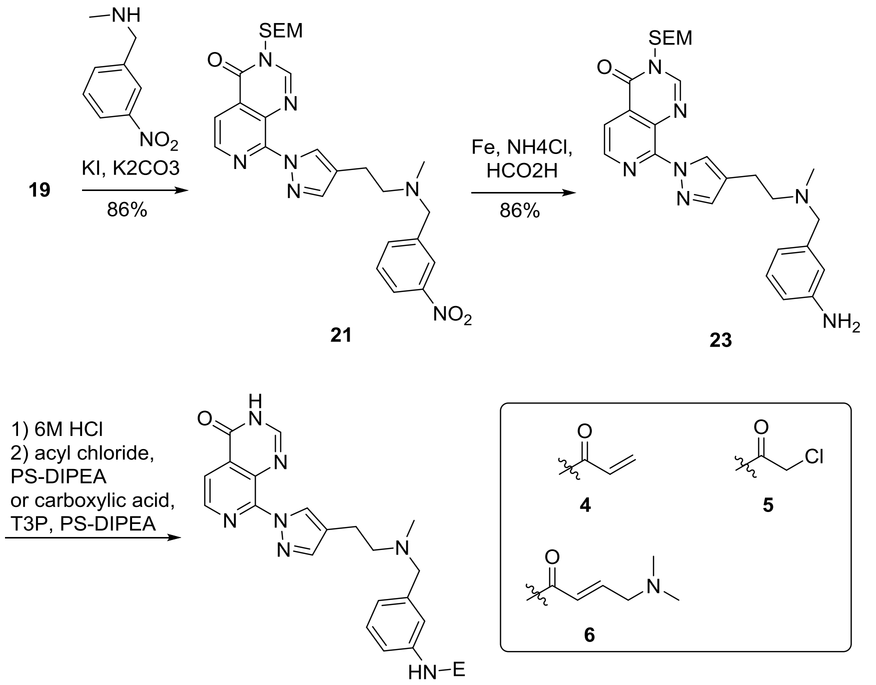

Scheme S2. Synthesis of methyl benzylamines 4-6

4-(2-((tert-butyldimethylsilyl)oxy)ethyl)-1H-pyrazole (17)

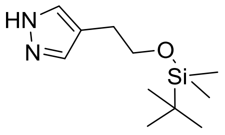

A solution of 2-(1H-pyrazol-4-yl)ethan-1-ol (1.000 g, 8.92 mmol) in anhydrous DMF (50 mL) was cooled to 0 °C and imidazole (0.911 g, 13.4 mmol) was added in one portion. After 15 min, *tert*-butylchlorodimethylsilane (1.479 g, 9.81 mmol) was added. The reaction was warmed to room temperature and stirred for 18 h. The solution was diluted with water (50 mL) and then extracted with Et_2O (75 mL). The organic layer was washed with water (50 mL) and brine (50 mL) and dried over Na_2SO_4. The filtrate was concentrated *in vacuo* (keeping the pressure above 450 mbar) to give **17** as a yellow oil (2.019 g, quant.) which was used without further purification. ^{1}H NMR (400 MHz, $CDCl_3$) δ 7.44 (s, 2H), 3.74 (t, J = 6.8 Hz, 2H), 2.70 (t, J = 6.8 Hz, 2H), 0.89 (s, 9H), -0.06 (s, 6H); LRMS (ESI$^+$) m/z 227.1 (M+H$^+$); HRMS (ESI$^+$): calcd for $C_{11}H_{22}N_2OSi^+$ [M+H]$^+$ 227.1574, found 227.1579.

tert-butyl 4-(2-(1-(4-oxo-3-((2-(trimethylsilyl)ethoxy)methyl)-3,4-dihydropyrido[3,4-d]pyrimidin-8-yl)-1H-pyrazol-4-yl)ethyl)piperazine-1-carboxylate (20)

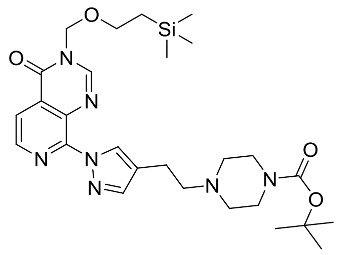

To a vial was added **19** (1.57 g, 3.37 mmol), *tert*-butyl piperazine-1-carboxylate (0.628 g, 3.37 mmol), potassium iodide (2.239 g, 13.49 mmol), potassium carbonate (9.32 g, 67.4 mmol) in MeCN (17 mL). The reaction mixture was heated at 75°C for 18 h. The reaction mixture was diluted with EtOAc (50 mL) and washed with $NaHCO_3$ (50 mL) and brine (50 mL). The organic layer was dried over Na_2SO_4, filtered and concentrated *in vacuo* to give an oil. The crude product was adsorbed on silica and purified by flash chromatography eluting with 0-10% MeOH in CH_2Cl_2 to give **20** as an off-white

solid (1.382 g, 74%). ^{1}H NMR (400 MHz, $CDCl_3$) δ 8.62 (d, *J* = 5.1 Hz, 1H), 8.56 (d, *J* = 0.8 Hz, 1H), 8.30 (s, 1H), 8.06 (d, *J* = 5.1 Hz, 1H), 7.78 (d, *J* = 0.8 Hz, 1H), 5.46 (s, 2H), 3.72 – 3.64 (m, 2H), 3.47 (t, *J* = 5.1 Hz, 4H), 2.79 (t, *J* = 7.6 Hz, 2H), 2.65 (t, *J* = 7.7 Hz, 2H), 2.48 (m, 4H), 1.47 (s, 9H), 1.18 – 0.84 (m, 2H), 0.00 (s, 9H); ^{13}C NMR (101 MHz, DMSO) δ 159.0, 153.8, 149.2, 148.1, 144.8, 141.9, 135.1, 131.1, 130.0, 120.6, 118.2, 78.7, 74.9, 66.4, 58.5, 52.4, 44.1, 28.1, 21.2, 17.4, -1.3; LRMS (ESI$^+$) m/z 556.37 (M+H$^+$); HRMS (ESI$^+$): calcd for $C_{27}H_{41}N_7O_4Si^+$ [M+H]$^+$ 556.3062, found 556.3077.

8-(4-(2-(piperazin-1-yl)ethyl)-1H-pyrazol-1-yl)pyrido[3,4-d]pyrimidin-4(3H)-one, HCl salt, (21)

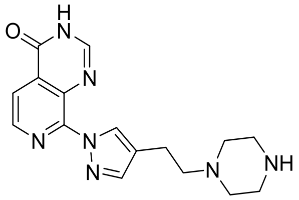

To a solution of **20** (63 mg, 0.113 mmol) in THF (1 mL) was added 6M HCl (1.134 mL, 6.80 mmol) and the solution was heated at 50 °C for 5 h. The solution was concentrated *in vacuo* to give **21** as a white solid (37 mg, quant.). Mp 241 – 245 °C; ^{1}H NMR (400 MHz, DMSO-d_6) δ 12.18 (s, 1H), 9.98 (s, 2H), 8.57 (d, *J* = 5.1 Hz, 1H), 8.51 (s, 1H), 8.33 (s, 1H), 8.01 (d, *J* = 5.1 Hz, 1H), 7.81 (s, 1H), 3.86 – 3.68 (s, 2H), 3.65 – 3.52 (m, 2H), 3.49 – 3.23 (m, 6H), 3.07 (dd, *J* = 10.6, 6.2 Hz, 2H); ^{13}C NMR (101 MHz, DMSO-d_6) δ 159.4, 147.7, 147.3, 144.3, 141.5, 135.5, 131.6, 130.8, 118.3, 117.2, 60.8, 55.4, 47.7, 18.4; LRMS (ESI$^+$) m/z 326.1 (M+H$^+$); HRMS (ESI$^+$): calcd for $C_{16}H_{19}N_7O^+$ [M+H]$^+$ 326.1715, found 326.1724.

8-(4-(2-(4-acryloylpiperazin-1-yl)ethyl)-1H-pyrazol-1-yl)pyrido[3,4-d]pyrimidin-4(3H)-one, (3)

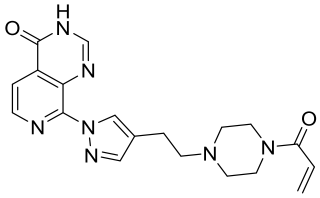

To a solution of **21** (150 mg, 0.377 mmol) in NMP (2 mL) was added PS-DIPEA (300 mg) and stirred for 5 min. The reaction mixture was cooled to 0 °C and acryloyl chloride (0.046 mL, 0.565 mmol) was added. The reaction mixture was warmed to room temperature and then stirred for 18 h. The solution was cooled to 0 °C and then more acryloyl chloride (56.2 µl, 0.692 mmol) was added. The reaction was warmed to room temperature and stirred for a further 6 h. The reaction mixture was filtered and washed with MeOH. The solution was concentrated *in vacuo* and then dried in a Genevac oven to give an orange solid. The crude product was adsorbed on silica and purified by flash chromatography eluting with 0-10% MeOH in CH_2Cl_2 to give **3** as a white solid (15 mg, 11%). ^{1}H NMR (400 MHz, DMSO-d_6) δ 8.53 (d, *J* = 5.1 Hz, 1H), 8.42 (d, *J* = 8.4 Hz, 1H), 8.28 (s, 1H), 7.97 (d, *J* = 5.1 Hz, 1H), 7.70 (d, *J* = 8.4 Hz, 1H), 6.80 (dd, *J* = 16.7, 10.5 Hz, 1H), 6.10 (dd, *J* = 16.7, 2.4 Hz, 1H), 5.67 (dd, *J* = 10.5, 2.4 Hz, 1H), 3.62 – 3.50 (m, 4H), 2.74 – 2.62 (m, 2H), 2.61 – 2.51 (m, 2H), 2.44 (q, *J* = 5.1, 4.4 Hz, 4H); ^{13}C NMR (101 MHz, DMSO-d_6) δ 164.2, 160.1, 147.9, 147.7, 143.9, 141.7, 131.1, 130.7, 128.3, 127.3, 120.4, 117.9, 58.4, 53.1, 45.1, 41.5, 21.2; LRMS (ESI$^+$) m/z 380.1 (M+H$^+$); HRMS (ESI$^+$): calcd for $C_{16}H_{19}N_7O^+$ [M+H]$^+$ 380.1829, found 380.1825.

8-(4-(2-(methyl(3-nitrobenzyl)amino)ethyl)-1H-pyrazol-1-yl)-3-((2-(trimethylsilyl)ethoxy)methyl)pyrido[3,4-d]pyrimidin-4(3H)-one (22)

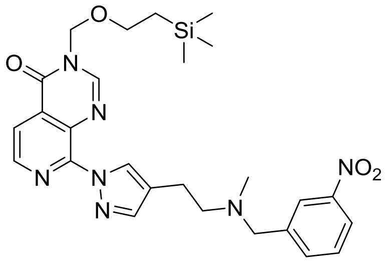

To a solution of **19** (1 g, 2.148 mmol), potassium iodide (1.426 g, 8.59 mmol) and potassium carbonate (5.94 g, 43.0 mmol) in ACN (21.48 mL) was added *N*-methyl-1-(3-nitrophenyl)methanamine (0.357 g, 2.15 mmol). The reaction mixture was stirred at 75 °C for 16 h. $NaHCO_3$ (60 mL) was added and back-extracted with EtOAc (2 x 75 mL). The combined organic extracts were washed with brine (75 mL). The organic layer was dried over Na_2SO_4, filtered and concentrated *in vacuo*. The crude product was adsorbed on silica and purified by flash chromatography eluting with 0-10% MeOH in CH_2Cl_2 to give **22** (0.985 g, 86%) as a yellow oil. ^1H NMR (400 MHz, $CDCl_3$) δ 8.63 (d, *J* = 5.1 Hz, 1H), 8.56 (s, 1H), 8.29 (s, 1H), 8.20 (s, 1H), 8.08 (m, 2H), 7.67 (d, *J* = 7.6 Hz, 1H), 7.46 (t, *J* = 7.9 Hz, 1H), 5.47 (s, 2H), 3.74 – 3.63 (m, 4H), 2.81 (t, *J* = 7.3 Hz, 2H), 2.76 – 2.68 (m, 2H), 2.31 (s, 3H), 1.02 – 0.93 (m, 2H), 0.00 (s, 9H); ^{13}C NMR (101 MHz, $CDCl_3$) δ 159.6, 147.1, 145.6, 143.3, 135.1, 134.7, 130.8, 129.3, 123.7, 122.3, 121.6, 118.1, 75.1, 68.1, 61.7, 58.3, 42.2, 22.7, 18.2, -1.3; LRMS (ESI^+) m/z 536.3 ($M+H^+$); HRMS (ESI^+): calcd for $C_{26}H_{33}N_7O_4Si^+$ $[M+H]^+$ 536.2436, found 536.2450.

8-(4-(2-((3-aminobenzyl)(methyl)amino)ethyl)-1H-pyrazol-1-yl)-3-((2-(trimethylsilyl)ethoxy)methyl)pyrido[3,4-d]pyrimidin-4(3H)-one (23)

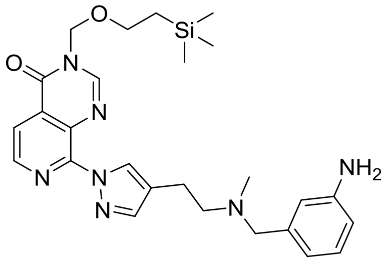

To a vial was added **22** (221 mg, 0.413 mmol) in 2-Propanol (1 mL) and water (0.3 mL). Iron (138 mg, 2.48 mmol) and ammonium chloride (883 mg, 16.5 mmol) was added. The reaction mixture was heated to 50 °C for 4 h. The reaction mixture was filtered through celite and washed with MeOH (50 mL). The filtrate was concentrated *in vacuo* to give a brown solid. The crude product was loaded onto an SCX-2 column and washed with MeOH. The product was eluted by washing with 3.5N NH_3 in MeOH and the solution was concentrated *in vacuo* to give **23** (179 mg, 86%) as a yellow amorphous solid. ^1H NMR (400 MHz, $CDCl_3$) δ 8.62 (d, *J* = 5.2 Hz, 1H), 8.55 (s, 1H), 8.29 (s, 1H), 8.05 (d, *J* = 5.1 Hz, 1H), 7.76 (s, 1H), 7.07 (t, *J* = 7.7 Hz, 1H), 6.72 – 6.64 (m, 2H), 6.60 – 6.52 (m, 1H), 5.46 (s, 2H), 3.73 – 3.64 (m, 2H), 3.50 (s, 2H), 2.80 (t, *J* = 7.4 Hz, 2H), 2.69 (t, *J* = 7.4 Hz, 2H), 2.30 (s, 3H), 1.02 – 0.93 (m, 2H), 0.00 (s, 9H); ^{13}C NMR (101 MHz, $CDCl_3$) δ 159.7, 148.4, 147.1, 146.6, 145.6, 143.5, 140.2, 134.7, 130.9, 130.4, 129.2, 121.9, 119.4, 118.0, 115.7, 114.0, 75.1, 68.1, 62.4, 58.0, 42.2, 22.5, 18.2, -1.3; LRMS (ESI^+) m/z 506.3 ($M+H^+$); HRMS (ESI^+): calcd for $C_{26}H_{35}N_7O_2Si^+$ $[M+H]^+$ 506.2694, found 506.2710.

N-(3-((methyl(2-(1-(4-oxo-3,4-dihydropyrido[3,4-d]pyrimidin-8-yl)-1H-pyrazol-4-yl)ethyl)amino)methyl)phenyl)acrylamide (4)

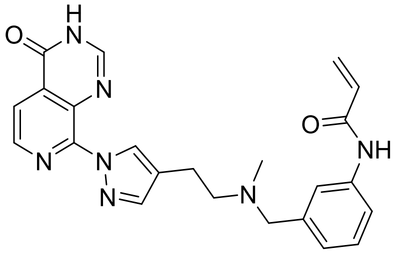

To a solution of **21** (0.179 g, 0.354 mmol) in THF (4 mL) was added HCl (4 mL, 24.00 mmol). The reaction mixture was heated to 50 °C for 3 hours. The reaction mixture was concentrated *in vacuo* to give 8-(4-(2-((3-aminobenzyl)(methyl)amino)ethyl)-1H-pyrazol-1-yl)pyrido[3,4-d]pyrimidin-4(3H)-one as the HCl salt (quant.). To a solution of 8-(4-(2-((3-aminobenzyl)(methyl)amino)ethyl)-1H-pyrazol-1-yl)pyrido[3,4-d]pyrimidin-4(3H)-one (0.075 g, 0.200 mmol) and PS-DIPEA (0.300 g) in DMA (2 mL) at 0 °C was added acryloyl chloride (0.024 mL, 0.300 mmol). The reaction mixture was warmed to room temperature and stirred for 3 h. The reaction mixture was filtered, washed with MeOH (40 mL) and concentrated *in vacuo*. The crude product was adsorbed on silica and purified by flash chromatography eluting with 25% MeOH in CH_2Cl_2 to give **4** (12 mg, 14%) as a white solid. ^{1}H NMR (400 MHz, DMSO-d_6) δ 10.08 (s, 1H), 8.52 (d, *J* = 5.1 Hz, 1H), 8.40 (s, 1H), 8.26 (s, 1H), 7.96 (d, *J* = 5.1 Hz, 1H), 7.69 (s, 1H), 7.64 – 7.55 (m, 2H), 7.24 (t, *J* = 7.8 Hz, 1H), 7.01 (d, *J* = 7.5 Hz, 1H), 6.40 (dd, *J* = 17.0, 10.1 Hz, 1H), 6.21 (dd, *J* = 17.0, 2.1 Hz, 1H), 5.72 (dd, *J* = 10.1, 2.1 Hz, 1H), 3.52 (s, 2H), 2.77 – 2.69 (m, 2H), 2.66 – 2.58 (m, 2H), 2.20 (s, 3H); ^{13}C NMR (101 MHz, DMSO) δ 163.0, 160.3, 148.1, 147.7, 143.7, 141.6, 139.8, 138.9, 136.0, 131.9, 131.1, 130.6, 128.5, 126.7, 123.9, 120.4, 119.5, 117.9, 61.5, 57.8, 41.7, 21.7; LRMS (ESI$^+$) m/z 430.2 (M+H$^+$); HRMS (ESI$^+$): calcd for $C_{23}H_{23}N_7O_2^+$ [M+H]$^+$ 430.1986, found 430.1986.

2-chloro-N-(3-((methyl(2-(1-(4-oxo-3,4-dihydropyrido[3,4-d]pyrimidin-8-yl)-1H-pyrazol-4-yl)ethyl)amino)methyl)phenyl)acetamide (5)

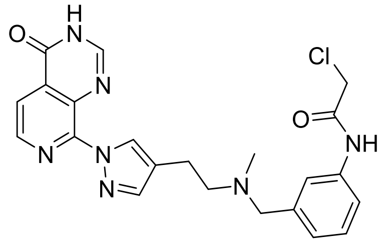

To a solution of **23** (100 mg, 0.266 mmol) and PS-DIPEA (300 mg) in NMP (2.5 mL) at 0 °C was added 2-chloroacetyl chloride (23 µl, 0.293 mmol). The reaction mixture was slowly warmed to room temperature and stirred for 16 h. The reaction mixture was filtered, washed with MeOH (50 mL) and concentrated *in vacuo* to give a brown oil. The crude product was adsorbed on silica and purified by flash chromatography eluting with 0-20% MeOH in CH_2Cl_2 to give **5** (0.045 g, 37%) as a white solid. ^{1}H NMR (400 MHz, DMSO-d_6) δ 10.26 (s, 1H), 8.55 (d, *J* = 5.2 Hz, 1H), 8.38 (s, 1H), 8.26 (s, 1H), 7.98 (d, *J* = 5.1 Hz, 1H), 7.69 (s, 1H), 7.55 (s, 1H), 7.48 (d, *J* = 8.3 Hz, 1H), 7.26 (t, *J* = 7.8 Hz, 1H), 7.03 (d, *J* = 7.6 Hz, 1H), 4.21 (s, 2H), 3.53 (s, 2H), 2.72 (d, *J* = 7.7 Hz), 2.63 (d, *J* = 7.8 Hz, 2H), 2.20 (s, 3H); ^{13}C NMR (101 MHz, DMSO) δ 164.5, 159.8, 148.0, 147.2, 144.1, 141.6, 138.4, 135.9, 131.1, 130.8, 128.7, 124.5, 119.7, 118.1, 117.9, 61.1, 57.7, 43.6, 41.4, 21.5; LRMS (ESI$^+$) m/z 452.7 (M+H$^+$); HRMS (ESI$^+$): calcd for $C_{22}H_{22}ClN_7O_2^+$ [M+H]$^+$ 452.1596, found 452.1566.

(E)-4-(dimethylamino)-N-(3-((methyl(2-(1-(4-oxo-3,4-dihydropyrido[3,4-d]pyrimidin-8-yl)-1H-pyrazol-4-yl)ethyl)amino)methyl)phenyl)but-2-enamide (6)

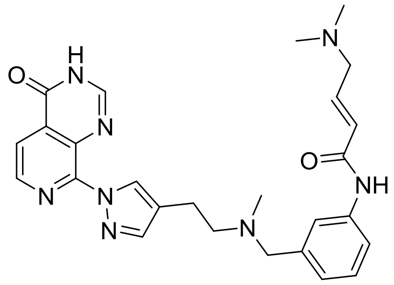

To a solution of **23** (100 mg, 0.266 mmol), PS-DIPEA (300 mg) and (*E*)-4-(dimethylamino)but-2-enoic acid, HCl (44 mg, 0.266 mmol) in DMA (2 mL) at 0 °C was added 2,4,6-tripropyl-1,3,5,2,4,6-trioxatriphosphinane 2,4,6-trioxide (0.235 mL, 0.400 mmol). The reaction mixture was warmed to room temperature and stirred for 16 h. The reaction mixture was filtered, washed with MeOH (50 mL) and concentrated *in vacuo* to give a brown oil. The crude product was adsorbed on silica and purified by flash chromatography eluting with 0-30% MeOH in CH_2Cl_2 to give **6** (39 mg, 30%) as a white solid. ^{1}H NMR (400 MHz, DMSO-d_6) δ 9.99 (s, 1H), 8.48 (d, *J* = 5.1 Hz, 1H), 8.42 (s, 1H), 8.25 (s, 1H), 7.94 (d, *J* = 5.1 Hz, 1H), 7.68 (s, 1H), 7.61 (d, *J* = 1.9 Hz, 1H), 7.55 (dd, *J* = 8.1, 2.0 Hz, 1H), 7.23 (t, *J* = 7.8 Hz, 1H), 6.99 (d, *J* = 7.6 Hz, 1H), 6.68 (dt, *J* = 15.4, 5.9 Hz, 1H), 6.22 (dt, *J* = 15.3, 1.7 Hz, 1H), 3.51 (s, 2H), 3.03 (dd, *J* = 5.9, 1.6 Hz, 2H), 2.73 (m, 2H), 2.62 (m, 2H), 2.19 (s, 3H), 2.16 (s, 6H); ^{13}C NMR (101 MHz, DMSO) δ 163.1, 159.8, 148.5, 147.5, 144.0, 141.6, 141.1, 139.7, 139.1, 135.8, 131.1, 130.7, 128.4, 126.0, 123.7, 120.5, 119.5, 117.8, 61.9, 61.5, 59.7, 57.7, 45.1, 41.7, 21.7; LRMS (ESI$^+$) m/z 487.6 (M+H$^+$); HRMS (ESI$^+$): calcd for $C_{26}H_{30}N_8O_2^+$ [M+H]$^+$ 487.2565, found 487.2569.

General synthetic procedures: Synthesis of PZ series

For the synthesis of the PZ derivatives, two main synthetic schemes are outlined in Scheme 3 and in Scheme 4. Intermediate **28** was synthesized according to Scheme 3 described in a literature procedure and its physical and spectroscopic properties are in agreement to those reported.[2]

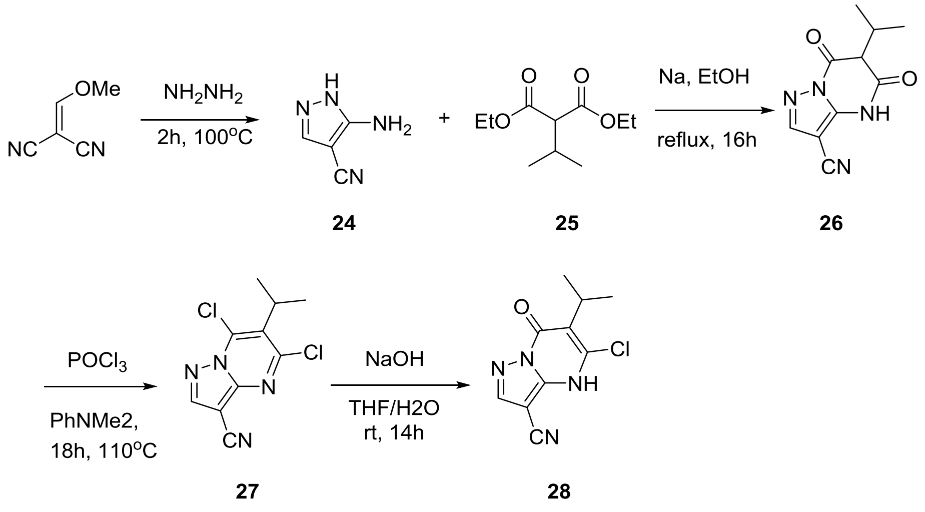

Scheme S3 Synthetic procedure for the preparation of intermediate 28

Synthesis of the final covalent KDOPZ derivatives is outlined in Scheme 4 General synthetic procedures in the route are described below.

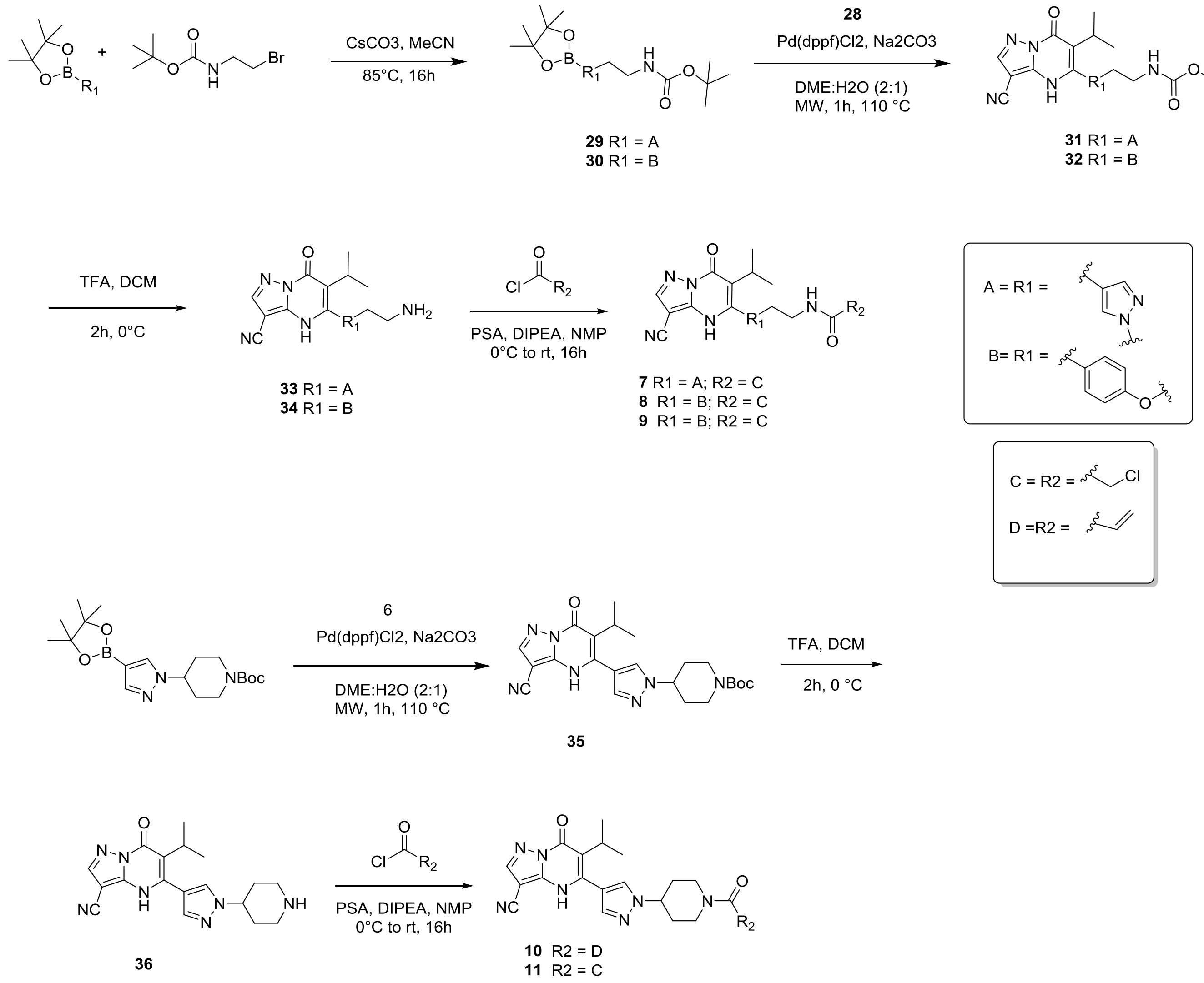

Scheme S4 Synthesis of PZ covalent inhibitors 7-11.

General procedure I: Synthesis of modified boronic esters (29 and 30)

The appropriate boronic ester (1 eq), cesium carbonate (2 eq.) and tert-butyl (2-bromoethyl)carbamate (1.5 eq) were suspended in Acetonitrile and the mixture was refluxed for 16h. After completion, reaction was cooled to room temperature, diluted with Et_2O and filtered. The filtrate was concentrated in vacuo and the crude was purified in a DCM:MeOH gradient from 0-5% MeOH to give the desired product.

General procedure II: Suzuki Coupling (31, 32 and 35).

5-chloro-6-isopropyl-7-oxo-4,7-dihydropyrazolo[1,5-a]pyrimidine-3-carbonitrile (**28**) (1 eq.), the appropriate aryl boronic ester (1.1 eq) , sodium carbonate (2 eq.) and Pd(dppf)Cl_2 (0.1 eq) were charged in a MW vial. The vial was sealed, evacuated and backfilled with nitrogen three times. A mixture of DME:Water (2:1) was added with a syringe and three more cycles of vacuum/nitrogen were applied. The reaction mixture was heated at 110 °C for 90 min under microwave condition. After completion, reaction was cooled to room temperature, diluted with EtOAC and filtered over a plough of celite. The solvent was removed under vacuum and the crude was purified on a Biotage Isolera in a DCM-MeOH gradient from 0-5 % to give the desired product.

General procedure III: Boc deprotections (33, 34 and 36)

On an ice bath, the Boc-protected amine was solubilized in anhydrous DCM. A 2:1 ratio in volume DCM:TFA was added dropwise and the resulting mixture was stirred at 0 °C for 2h. The solvent was removed in vacuo and the crude was solubilized in MeOH and flushed through a SCX-2 isolute column. The cartidge was first washed with 2CV of methanol and then compound was eluted flushing with 1N amonia in methanol to get the pure product as a brown solid.

General procedure IV: Final acylation step (7-11)

To a solution of the deprotected amine (1 eq) in dry N-Methyl-2-pyrrolidinone (NMP) was added DIPEA-polystyrene (4 eq) previously washed in MeOH and dried. The reaction mixture was cooled at 0 °C and the appropriate acid chloride (1.5 eq) was added dropwise. The reaction mixture was then left to warm up to room temperature overnight. After completion, the reaction mixture was filtered and washed with MeOH. The solution was concentrated in vacuum and then purified by HPLC to obtain the final product as solid.

Example of synthesis of modified boronic ester.

tert-butyl (2-(4-(4,4,5,5-tetramethyl-1,3,2-dioxaborolan-2-yl)-1H-pyrazol-1 yl)ethyl)carbamate (29)

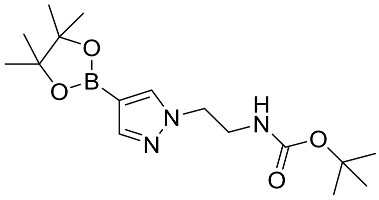

4-(4,4,5,5-tetramethyl-1,3,2-dioxaborolan-2-yl)-1H-pyrazole (1.020 g, 5.15 mmol), cesium carbonate (3.36 g, 10.31 mmol) and tert-butyl (2-bromoethyl)carbamate (1.805 g, 7.73 mmol) were suspended in Acetonitrile (15 ml) and the mixture was refluxed for 16h. After completion, reaction was cooled to room temperature, diluted with Et2O and filtered. The filtrate was concentrated in vacuo and the crude was purified in a DCM:MeOH gradient from 0-5% MeOH to give the product as a white solid.

White solid, 92%, LCMS (ES+): RT 1.48 min, m/z 338 $[M+H]^+$, 281 $[M+H\text{-}t\text{-}Bu]^+$ ^1H NMR (400 MHz, DMSO-d6) δ 7.85 (s, 1H), 7.58 (s, 1H), 6.87 (t, J = 5.7 Hz, 1H), 4.14 (q, J = 8.0, 7.1 Hz, 2H), 3.30 (q, J = 6.0 Hz, 2H), 1.35 (s, 9H), 1.25 (s, 12H). ^{13}C NMR (101 MHz, DMSO) δ 156.0, 145.1, 137.6, 83.3, 78.3, 74.0, 51.0, 49.1, 28.7, 25.4, 25.1.

tert-butyl (2-(4-(4,4,5,5-tetramethyl-1,3,2-dioxaborolan-2-yl)phenoxy)ethyl)carbamate (30)

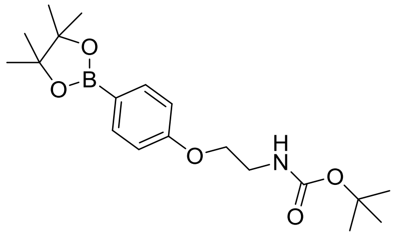

Synthesis as described in general procedure I.

Colorless oil, 65%, LCMS (ES+): RT 1.85 min, m/z 364 $[M+H]^+$, 307 $[M+H\text{-}t\text{-}Bu]^+$. ^1H NMR (400 MHz, Chloroform-*d*) δ 7.7 (d, *J* = 8.6 Hz, 2H), 6.88 (d, *J* = 8.6 Hz, 2H), 5.00 (s, 1H), 4.04 (t, *J* = 5.1 Hz, 2H), 3.53 (q, *J* = 5.5 Hz, 2H), 1.45 (s, 9H), 1.33 (s, 12H). ^{13}C NMR (101 MHz, $CDCl_3$) δ 161.1, 155.9, 136.6, 135.1, 113.9, 113.8, 83.6, 79.6, 67.0, 50.8, 40.1, 28.4, 24.9.

Example of Suzuki coupling

tert-butyl (2-(4-(3-cyano-6-isopropyl-7-oxo-4,7-dihydropyrazolo[1,5-a]pyrimidin-5-yl)-1H-pyrazol-1-yl)ethyl)carbamate (31)

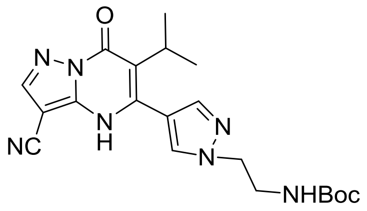

5-chloro-6-isopropyl-7-oxo-4,7-dihydropyrazolo[1,5-a]pyrimidine-3-carbonitrile (580 mg, 2.451 mmol), tert-butyl (2-(4-(4,4,5,5-tetramethyl-1,3,2-dioxaborolan-2-yl)-1H-pyrazol-1-yl)ethyl)carbamate (909 mg, 2.70 mmol) , sodium carbonate (520 mg, 4.90 mmol) and Pd(dppf)Cl2 (180 mg, 0.245 mmol) were charged in a MW vial. The vial was sealed, evacuated and backfilled with nitrogen few times. A mixture of DME:Water (2:1) was added with a syringe and few more cycles of vacuum/nitrogen were applied. The reaction mixture was heated at 110 °C for 90 min under microwave condition. After completion, reaction was cooled to room temperature, diluted with EtOAC and filtered over a plough of celite. The solvent was removed under vacuum and the crude was purified on a Biotage in a DCM-MeOH gradient from 0-5 to give the desired product.

Brown solid, 86%, LCMS (ES+): RT 1.22 min, m/z 412 $[M+H]^+$, 356 $[M+H\text{-}t\text{-}Bu]^+$ ^{1}H NMR (400 MHz, DMSO-d_6) δ 13.05 (s, 1H), 8.28 (s, 1H), 8.06 (s, 1H), 7.73 (s, 1H), 6.96 (t, J = 5.7 Hz, 1H), 4.23 (t, J = 6.4 Hz, 2H), 3.41 – 3.37 (m, 2H), 3.04 (p, J = 6.9 Hz, 1H), 1.36 (s, 9H), 1.30 (d, J = 6.9 Hz, 6H). ^{13}C NMR (101 MHz, DMSO) δ 172.5, 156.0, 145.5, 139.5, 131.7, 78.4, 74.4, 51.4, 49.1, 28.7, 28.6, 21.5, 20.8.

tert-butyl (2-(4-(3-cyano-6-isopropyl-7-oxo-4,7-dihydropyrazolo[1,5-a]pyrimidin-5-yl)phenoxy)ethyl)carbamate (32)

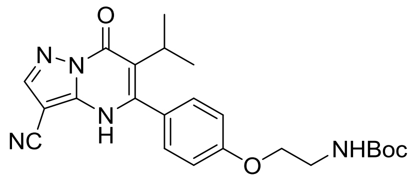

Synthesis as described in general procedure II. Crude was used without further purification into the next step. LCMS (ES+): RT 1.42 min, m/z 438 $[M+H]^+$, 382 $[M+H\text{-}t\text{-}Bu]^+$.

tert-butyl 4-(4-(3-cyano-6-isopropyl-7-oxo-4,7-dihydropyrazolo[1,5-a]pyrimidin-5-yl)-1H-pyrazol-1-yl)piperidine-1-carboxylate (35)

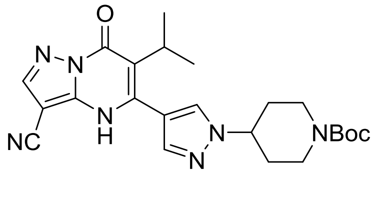

Synthesis as described in general procedure II

Brown solid, 63%, LCMS (ES+): RT 1.35 min, m/z 450 $[M+H]^+$, 407 $[M+H\text{-}t\text{-}Bu]^+$. ^{1}H NMR (400 MHz, DMSO-d_6) δ 13.05 (s, 1H), 8.36 (s, 1H), 8.22 (s, 1H), 7.76 (s, 1H), 4.50 (tt, J = 11.3, 4.0 Hz, 1H), 4.07 (d, J = 13.2 Hz, 2H), 3.05 – 2.82 (m, 3H), 2.06 (d, J = 13.2 Hz, 2H), 1.85 (qd, J = 12.3, 4.4 Hz, 2H), 1.42 (s, 9H), 1.29 (d, J = 6.9 Hz, 6H). ^{13}C NMR (101 MHz, DMSO) δ 155.0, 153.9, 145.3, 138.6, 129.4, 113.8, 112.9, 78.9, 73.9, 58.4, 31.9, 28.3, 28.1, 20.2.

Example of Boc-deprotection

5-(1-(2-aminoethyl)-1H-pyrazol-4-yl)-6-isopropyl-7-oxo-4,7-dihydropyrazolo[1,5-a]pyrimidine-3-carbonitrile (33)

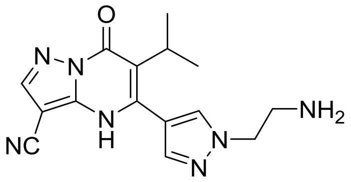

On an ice bath, tert-butyl (2-(4-(3-cyano-6-isopropyl-7-oxo-4,7-dihydropyrazolo[1,5-a]pyrimidin-5-yl)-1H-pyrazol-1-yl)ethyl)carbamate (485 mg, 1.179 mmol) was suspended in anhydrous DCM (4 ml). A volume of TFA (3.08 ml) was added dropwise and the resulting mixture was stirred at 0 °C for 2h. The solvent was removed in vacuo and the crude was solubilized in MeOH, flushed through a SCX-2 isolute column. The cartidge was washed with 2CV of methanol and then compound was eluted flushing with 7N amonia in methanol to get the pure product as a brown solid.

White solid, 78%, LCMS (ES+): RT 1.00 min, m/z 312 $[M+H]^+$. ^{1}H NMR (400 MHz, DMSO-d_6) δ 8.02 (s, 1H), 8.00 (s, 1H), 7.69 (s, 1H), 4.43 (t, *J* = 6.1 Hz, 2H), 3.35 (t, *J* = 6.1 Hz, 2H), 3.18 (p, *J* = 6.9 Hz, 1H), 1.34 (d, *J* = 6.9 Hz, 6H). ^{13}C NMR (101 MHz, DMSO-d_6) δ 157.5, 152.2, 151.1, 144.5, 139.8, 131.2, 123.1, 117.2, 111.1, 74.4, 49.1, 28.9, 21.2.

5-(4-(2-aminoethoxy)phenyl)-6-isopropyl-7-oxo-4,7-dihydropyrazolo[1,5-a]pyrimidine-3-carbonitrile (34)

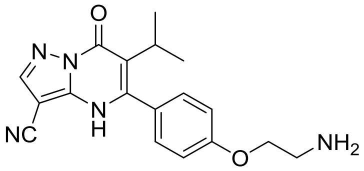

Synthesis as described in general procedure III
Light brown solid, 89%, LCMS (ES+): RT 1.06 min, m/z 338 $[M+H]^+$, ^{1}H NMR (400 MHz, DMSO-d_6) δ 8.06 (s, 1H), 7.31 (d, *J* = 8.5 Hz, 2H), 7.02 (d, *J* = 8.3 Hz, 2H), 4.23 (t, *J* = 5.0 Hz, 2H), 3.29 (t, *J* = 5.0 Hz, 2H), 3.23 (s, 2H), 2.79 (p, *J* = 6.8 Hz, 1H), 1.26 (d, *J* = 6.9 Hz, 6H). ^{13}C NMR (101 MHz, DMSO) δ 172.6, 159.2, 157.6, 152.1, 144.5, 135.6, 129.9, 117.0, 114.3, 111.3, 74.8, 65.1, 29.3, 21.1, 21.0.

6-isopropyl-7-oxo-5-(1-(piperidin-4-yl)-1H-pyrazol-4-yl)-4,7-dihydropyrazolo[1,5-a]pyrimidine-3-carbonitrile (36)

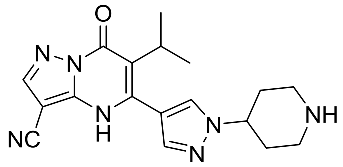

Synthesis as described in general procedure III
White solid, 91%, LCMS (ES+): RT 1.02 min, m/z 352 $[M+H]^+$. ^{1}H NMR (400 MHz, DMSO-d_6) δ 8.03 (s, 1H), 7.93 (s, 1H), 7.63 (s, 1H), 4.55 (tt, *J* = 10.0, 4.3 Hz, 1H), 3.44 (dt, *J* = 13.0, 3.8 Hz, 2H), 3.14 – 3.00 (m, 3H), 2.28 – 2.06 (m, 4H), 1.33 (d, *J* = 6.9 Hz, 6H). ^{13}C NMR (101 MHz, DMSO) δ 157.4, 153.4, 152.2, 151.2, 144.4, 138.9, 132.9, 128.6, 118.7, 74.21, 56.3, 43.5, 30.3, 29.0, 21.2.

Example of acylation

2-chloro-N-(2-(4-(3-cyano-6-isopropyl-7-oxo-4,7-dihydropyrazolo[1,5-a]pyrimidin-5-yl)-1H-pyrazol-1-yl)ethyl)acetamide (7)

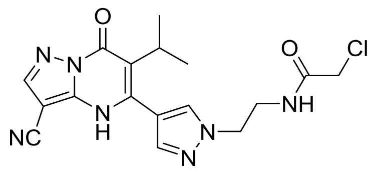

To a solution of 5-(1-(2-aminoethyl)-1H-pyrazol-4-yl)-6-isopropyl-7-oxo-4,7-dihydropyrazolo[1,5-a]pyrimidine-3-carbonitrile (51.0 mg, 0.164 mmol) in N-Methyl-2-pyrrolidinone (NMP) (2 ml) was added DIPEA-polystyrene (0.655 mmol) previously dried and washed. The reaction mixture was cooled at 0 °C and 2-chloroacetyl chloride chloride (0.020 ml, 0.246 mmol) was added dropwise. The reaction mixture was then left to cool down at room temperature overnight. The reaction mixture was filtered and washed with MeOH. The solution was concentrated in vacuum and then was purified by HPLC to obtain the final product as a white solid.

White solid, 55%, LCMS (ES+): RT 1.12 min, m/z 388 $[M+H]^+$, 390 $[M+H+2]^+$. ^{1}H NMR (400 MHz, DMSO-d6) δ 13.07 (s, 1H), 8.39 (t, J = 5.7 Hz, 1H), 8.34 (s, 1H), 8.11 (s, 1H), 7.78 (s, 1H), 4.31 (t, J = 6.3 Hz, 2H), 4.06 (s, 2H), 3.57 (q, J = 6.1 Hz, 2H), 3.01 (h, J = 6.9 Hz, 1H), 1.30 (d, J = 6.9 Hz, 6H). ^{13}C NMR (101 MHz, DMSO) δ 166.8, 155.5, 145.6, 139.8, 132.0, 114.1, 113.6, 109.8, 74.4, 50.9, 43.0, 28.7, 20.7.

2-chloro-N-(2-(4-(3-cyano-6-isopropyl-7-oxo-4,7-dihydropyrazolo[1,5-a]pyrimidin-5-yl)phenoxy)ethyl)acetamide (8)

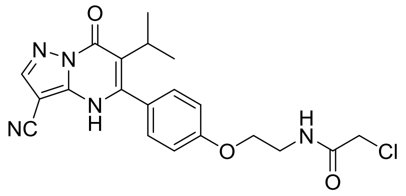

Synthesis as described in general procedure IV

Light yellow solid, 89%, LCMS (ES+): RT 1.21 min, m/z 414 $[M+H]^+$, 416 $[M+H+2]^+$. ^{1}H NMR (400 MHz, DMSO-d6) δ 13.33 (s, 1H), 8.51 (t, J = 5.6 Hz, 1H), 8.37 (s, 1H), 7.43 (d, J = 8.7 Hz, 2H), 7.13 (d, J = 8.7 Hz, 2H), 4.17 – 4.06 (m, 4H), 3.53 (q, J = 5.6 Hz, 2H), 2.67 (p, J = 6.9 Hz, 1H), 1.24 (d, J = 6.9 Hz, 6H). ^{13}C NMR (101 MHz, DMSO) δ 166.8, 159.9, 155.6, 145.7, 130.6, 114.9, 114.4, 113.4, 74.4, 66.7, 43.0, 39.7, 39.1, 28.8, 20.5.

N-(2-(4-(3-cyano-6-isopropyl-7-oxo-4,7-dihydropyrazolo[1,5-a]pyrimidin-5-yl)phenoxy)ethyl)acrylamide (9)

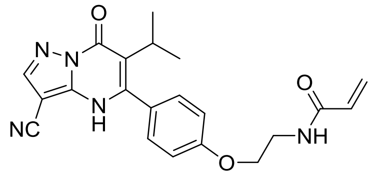

Synthesis as described in general procedure IV

Light yellow solid, 79%, LCMS (ES+): RT 1.23 min, m/z 392 $[M+H]^+$. ^{1}H NMR (400 MHz, DMSO-d6) δ 13.33 (s, 1H), 8.42 (t, J = 5.7 Hz, 1H), 8.37 (s, 1H), 7.43 (d, J = 8.6 Hz, 2H), 7.13 (d, J = 8.6 Hz, 2H), 6.28 (dd, J = 17.1, 10.1 Hz, 1H), 6.12 (dd, J = 17.1, 2.3 Hz, 1H), 5.62 (dd, J = 10.1, 2.3 Hz, 1H), 4.13 (t, J = 5.6 Hz, 2H), 3.56 (q, J = 5.6 Hz, 2H), 2.67 (p, J = 6.1 Hz, 1H), 1.23 (d, J = 6.9 Hz, 6H). ^{13}C NMR (101 MHz, DMSO) δ 165.4, 160.0, 155.6, 145.7, 132.0, 130.6, 125.9, 114.9, 74.4, 66.9, 40.6, 38.7, 28.8, 20.5.

5-(1-(1-acryloylpiperidin-4-yl)-1H-pyrazol-4-yl)-6-isopropyl-7-oxo-4,7-dihydropyrazolo[1,5-a]pyrimidine-3-carbonitrile (10)

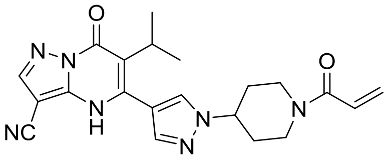

Synthesis as described in general procedure IV

White solid, 62%, LCMS (ES+): RT 1.14 min, m/z 406 $[M+H]^+$. ^1H NMR (400 MHz, DMSO-d_6) δ 13.05 (s, 1H), 8.24 (s, 1H), 8.14 (s, 1H), 7.71 (s, 1H), 6.87 (dd, *J* = 16.7, 10.5 Hz, 1H), 6.13 (dd, *J* = 16.7, 2.4 Hz, 1H), 5.70 (dd, *J* = 10.5, 2.4 Hz, 1H), 4.58 (tt, *J* = 11.5, 4.2 Hz, 2H), 4.20 (d, *J* = 13.9 Hz, 1H), 3.24 (d, *J* = 13.1 Hz, 1H), 3.02 (p, *J* = 6.9 Hz, 1H), 2.84 (d, *J* = 12.8 Hz, 1H), 2.12 (d, *J* = 11.3 Hz, 2H), 2.01 – 1.73 (m, 3H), 1.30 (d, *J* = 6.9 Hz, 6H).

5-(1-(1-(2-chloroacetyl)piperidin-4-yl)-1H-pyrazol-4-yl)-6-isopropyl-7-oxo-4,7-dihydropyrazolo[1,5-a]pyrimidine-3-carbonitrile (11)

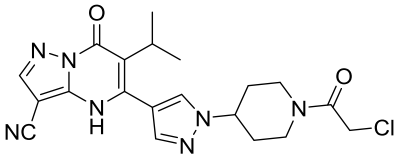

Synthesis as described in general procedure IV

White solid, 57%, LCMS (ES+): RT 1.17 min, m/z 428 $[M+H]^+$, 430 $[M+H+2]^+$. ^1H NMR (400 MHz, DMSO-d_6) δ 13.06 (s, 1H), 8.32 (s, 1H), 8.18 (s, 1H), 7.75 (s, 1H), 4.67 – 4.51 (m, 1H), 4.48 – 4.36 (m, 3H), 3.98 (d, *J* = 13.9 Hz, 1H), 3.26 (t, *J* = 12.8 Hz, 1H), 3.00 (p, *J* = 6.9 Hz, 1H), 2.86 (t, *J* = 12.6 Hz, 1H), 2.19 – 1.74 (m, 4H), 1.30 (d, *J* = 6.9 Hz, 6H). ^{13}C NMR (101 MHz, DMSO) δ 170.5, 155.4, 145.8, 143.9, 141.2, 139.1, 129.9, 114.4, 113.3, 113.0, 74.4, 60.5, 58.7, 32.1, 28.7, 20.6.

Biological and Biochemical methods

Protein purification

A KDM5B construct encoding regions Phe26-Ile770 was amplified from an OriGene cDNA clone and cloned into a pFastBac-derived vector (pFB-LIC-Bse) containing a TEV protease-cleavable N-terminal 6x-histidine tag. The recombinant KDM5B (residues 26–770) construct was expressed in Sf9 cells, and generation of recombinant baculoviruses, insect cell culture, and infections was performed according to the manufacturer's instructions (Invitrogen) (Johansson et al., 2016).[3] The cells were harvested 72 hr post infection and lysed in a buffer containing 50 mM HEPES (pH 7.5), 500 mM NaCl, 10 mM imidazole, 5% glycerol, 0.5 mM Tris(2-carboxyethy1)phosphine, and a proteinase inhibitor cocktail (Calbiochem), and purified using nickel-affinity chromatography using a stepwise gradient of imidazole. The eluted protein was then incubated with TEV protease at 4°C overnight, followed by size-exclusion chromatography (Superdex 200). The TEV protease and uncleaved protein were removed using nickel-affinity chromatography and protein was concentrated to 8.1 mg/mL and stored at −80°C.

Protein Crystallisation, X-ray Data Collection and Structure Determination

Protein preparations were concentrated in Amicon concentrators to 7.5 mg/mL, and were subjected to crystallization experiments at 4°C using the sitting-drop vapor diffusion method. The protein was pre-incubated with 4 mM MnCl2 before the protein was transferred to crystallization plates. KDM5B yielded diffracting crystals in a drop consisting of 50 nL protein-compound mix (7.5 mg/mL) and 100 nL of a precipitant consisting of 1.6 M Na/K phosphate, 0.1 M HEPES

(pH 7.5), and 20 nL of KDM5B seeds of crystals obtained from the same condition. Then the crystals were soaked with 5mM of each compound for 24 hours at 4°C and Crystal was cryoprotected with mother liquor supplemented with 25% ethylene glycol before they were flash frozen in liquid nitrogen. The datasets were collected on beamline I04 at the Diamond Light Source. The crystals diffracted to between 2.0 and 2.15 Å, and the dataset was processed in Xia2 with spacegroup P6122 and reindexed to P6522 and scaled and merged with Aimless. The structure was refined with a KDM5B model (51af). The protein-ligand model was further improved by subsequent cycles of model building in Coot and refinement with Phenix. The ligand library was generated with ELBOW. The quality of the structure was assessed with MolProbity server.

KDM alpha screen selectivity

Inhibition of histone demethylases was assessed using the histone demethylase AlphaScreen assay (Amplified Luminescence Proximity Homogenous Assay) as previously reported.[4] The basis of this assay for lysine demethylases have been widely described.[5] In brief, the assay was performed in 384-well plate format using white proxiplates (PerkinElmer), and transfer of titrations of compounds (100 nl) was performed using an ECHO 550 acoustic dispenser (Labcyte). Then, for IC50 determinations, 5 µl of assay buffer (50 mM HEPES pH 7.5, 0.1% (w/v) BSA and 0.01 % (v/v) Tween- 20) containing the appropriate demethylase enzyme (see Supplementary table 1 for final concentrations) was transferred to each well of a 384-well proxiplate with a Multidrop™ Combi Reagent Dispenser, and the enzymes allowed to pre-incubate for 120 minutes with compound (final concentration of DMSO was 1%). After that time, the enzyme reaction was initiated by addition of 5 µl of a substrate mix consisting of Ketoglutarate (2-OG), Ferrous Ammonium Sulphate (FAS), L-Ascorbic Acid (LAA) and biotinylated peptide substrate (see Supporting table X for final assay concentrations) and the reaction incubated for the indicated time at room temperature. Reaction was stopped by the addition of 5 µl of assay buffer containing EDTA (7.5 mM) and NaCl (400 mM). Streptavidin Donor beads (0.08 mg/ml) and Protein-A conjugated acceptor beads (0.08 mg/ml) (AlphaScreen General IgG detection kit was from Perkin Elmer) were pre-incubated for 1 hour with an antibody to the product methyl mark (Supplementary Table 1) and the presence of biotin-H3-product was detected by addition of 5 µl of the pre-incubated AlphaScreen beads (final concentrations of 0.02 mg/ml with respect to acceptor and donor beads). Detection was allowed to proceed for 2 hour at room temperature and the assay plates read in a BMG Labtech Pherastar FS plate reader (excitation, 680 nm; emission, 570 nm). Data were normalized to the no enzyme control and the IC50 determined from the nonlinear regression curve fit using GraphPad Prism 7.

Timecourse KDM5B inhibition and Kinact/Ki calculations

The timecourse alphascreen assay was performed using the conditions described in the AlphaScreen section for KDM5B but with different pre-incubation times (from 0 to 120 min) for the enzyme with compound. Data were normalized to the no enzyme control and the IC50 for each timepoint was determined from the nonlinear regression curve fit using GraphPad Prism 7. The Ki and kinact were calculated through a least squares fit of the obtained IC50 at the different timepoints into the equation below which was derived by Krippendorff *et al*.[6] S is the H3K4me3 peptide substrate concentration of 0.1 µM and KM is the Michaelis-Menten constant for the substrate of 0.5 µM. XLFit software was used to compute the values of Ki and kinact.

$$\mathrm{IC_{50}(t)} = \mathrm{K}_i\left(1+\frac{\mathrm{S}}{\mathrm{K_M}}\right).\left(\frac{2-2e^{-\eta_{\mathrm{IC_{50}}}\cdot\, \mathrm{k_{inact}}\cdot\, \mathrm{t}}}{\eta_{\mathrm{IC_{50}}}\cdot\, \mathrm{k_{inact}}\cdot \mathrm{t}}-1\right) \quad (1)$$

$$\eta_{\mathrm{IC_{50}}} = \frac{\mathrm{IC_{50}(t)}}{\mathrm{K_i}\left(1+\frac{\mathrm{S}}{\mathrm{K_M}}\right)+\mathrm{IC_{50}(t)}} \quad (2)$$

KDM5B 2-OG competition assay

2-OG competition assay were performed as described for the general KDM5B AlphaScreen method above, varying the 2-OG concentrations from a range of 1mM to 0.5 µM. Data were normalized to the no enzyme control and the IC_{50} determined from the nonlinear regression curve fit using GraphPad Prism 7.

Covalent binding RapidFire

Recombinant KDM5B (MW = 55152 Da) was incubated at 1 μM with the appropriate compound at 10 μM for 2 hours at 37^0C in 50 mM MES buffer at pH 7.0. Samples were injected in RapidFire 365 and MS was detected in QTOF mass spectrometer. Data were acquire and analysed with Agilent Mass Hunter software.

Mapping

Recombinant KDM5B was incubated at 1 μM with the appropriate compound at 10 μM for 2 hours at 37^0C in 200 μL of 50 mM MES buffer at pH 7.0. Then 5 μl of the DTT reducing reagent were added (final concentration 5 mM) and vortex. Sample was incubated for 60 min at room temperature followed by addition of 20 μl of the iodoacetamide alkylating reagent (final concentration 20 mM) and vortex. The mixture was incubated for 60 min at room temperature and then the protein was precipitated via Methanol/Chloroform Extraction. The protein pellet was resuspended in 50 μl 6 M urea buffer by vortexing and sonication and then diluted with 250 μl MilliQ-H20 to a final concentration of < 1M urea. Digestion with elastase (1 μL) overnight at 37 ºC followed by purification with SEP-PAK C18 columns and resuspension of the final sample in 98% MilliQ-H20 2% CH3CN 0.1% TFA gave the final sample ready to inject. The samples were injected into an Orbitrap MS for LCMS/MS of peptide fragments and data analysis was performed with PEAKS 8.0

NanoLuciferase Bioluminescent Resonance Energy Transfer (NanoBRET™) Assay

HEK293 cells (4x105/well) were plated in 6-well plates and transfected with NanoLuciferase fusions of the KDM proteins and Transfection Carrier DNA (Promega UK) at ratios 1:10. Twenty hours post-transfection, the cells were collected, washed with PBS and resuspended in Opti-MEM without serum or phenol red. Cell density was adjusted to 2x105/mL and replated in 384-well white polystyrene cell culture microplates (Greiner Bio-One Ltd, UK).

To access tracer affinity, cells were treated with a concentration range of the tracer and with a fixed concentration of 20 μM of a known inhibitor or DMSO as a vehicle control. The plates were incubated for 3 hours at 37oC/5% CO2.

To access test compound potency, cells were treated with a concentration range of the test compounds, with a fixed tracer concentration of 2 μM or DMSO as a vehicle control. The plates were incubated for 3 hours at 37oC/5% CO2.

NanoBRET™ Nano-Glo® substrate (Promega UK) and Extracellular NanoLuc Inhibitor (Promega UK) were added at final concentrations of 10 and 20 μM, respectively. Readings were performed within 5 minutes using a PHERAstar FSX (BMG Labtech) with 450 and 610 nm filters. Corrected BRET ratios were calculated and is defined as the ratio if the emission at 610/450 nm for experimental samples (i.e. those treated with tracer) minus the emission at 610/450 nm for control samples (DMSO controls). BRET ratios are expressed as milliBRET units (mBU), where 1 mBU corresponds to the corrected BRET ratio multiplied by 1000. Data were analyzed using MS Excel 2010 and Prism 7 graphing software (GraphPad).

Cytotoxicity assessment

Cell Titer-Glo™ (Promega UK) luminescent cell viability assay was used to assess cytotoxicity after culturing HEK-293 cells in the presence of the test KDM5 inhibitors for 72 hours. Cells (2x103/well) were plated in 384-well plates with defined concentration ranges of the test compounds, to allow determination of IC_{50} values. Luminescent readings were measured using a PHERAstar FSX (BMG Labtech).

ChIP-seq

HEK cells were cultured at 37 °C and 5 % CO2 in DMEM–Dulbecco's Modified Eagle Medium (Gibco) and supplemented with 10 % FCS (Invitrogen) and 1 % Glutamax (Invitrogen). HEK293 cells were then treated with 2 μM of the appropriate compound or DMSO for 72h. Just before fixation in 1%formaldehyde, 15% of SF9 cells were added to each sample. Once human and SF9 cells were combined, the sample was treated as a single ChIP-seq sample throught the experiment. Cells were fixed for 8 min at room temperature, lysed, and sonicated at 4ºC on a Bioruptor Pico for 4 repeats of 10 cycles of 30 s on/30 s off. The sonicated lysates were incubated for 1 hr at 4ºC with pre-blocked 1:1 protein A: protein G dynabeads, prior to overnight incubation with 1 mg of anti-H3K4me3 antibody (Millipore

cat no. 07–473 lot no. 2930138). Pre- blocked beads were added for 1 hr, the beads washed, and DNA was eluted followed by Proteinase K digestion. Libraries were constructed using the NEBNext UltraTM DNA library prep kit (NEB) and sequenced on an Illumina NextSeq 500 platform. Libraries were also generated for the input of each condition and sequenced in a similar manner.

Data Processing of ChIP-Seq

Sequences were demiltiplexed and reads were aligned to the concatenated human/SF9 genome generated earlier using Bowtie 2 (version 2.3.4.2), allowing only reads that matched uniquely to one location. The number of reads mapping to the human and SF9 genomes separately in each condition was calculated. The number of SF9 reads-per-million total aligned reads was calculated for each pull-down condition and corrected according to the percentage of SF9 reads in the corresponding input samples. A scaling factor for normalization was then calculated for each pull-down based on the reciprocal of the corrected SF9 reads per million for each sample. The aligned Bam files were converted to bedGraph files using BedTools for each strand of each sample using the scaling factor derived earlier. Homer v4.8 was used to read the produced bedGraphs for each sample and create a Tag Directory for each sample that was used for subsequent calculations of coverage. Coverage at the transcription start site and across each peak was calculated for each condition using Homer for both reads normalized on a reads-per- million basis and those normalized with the Sf9 spike in and an average taken to produce the values plotted. Data analysis was performed using CGAT tools and pipelines.[7]

Statistical analysis of data.

Statistical analysis of the structural and the sequencing data was automatically performed in all of the programs used as noted above. Other statistical analyses were performed using the GraphPad Prism 7 software.

Supplementary Figures and Tables

Table S1 k_{inact} and K_i values for compounds 3-11

cmpd	K_i (nM)	k_{inact} (10^{-4}/s)	k_{inact}/K_i (10^3/sM) (n)
**3**	834 ± 79	12 ± 0.13	1.4 ± 0.02 (2)
**4**	289 ± 30	21 ± 0.21	7.4 ± 0.04 (2)
**5**	536 ± 140	20 ± 3.5	40 ± 17 (4)
**6**	124 ± 6.0	7.6 ± 0.51	6.1 ± 0.12 (2)
**7**	226 ± 37	56 ± 9.6	25 ± 0.20 (2)
**8**	691 ± 13	48 ± 1.7	6.9 ± 0.11 (2)
**9**	723 ± 69	42 ± 4.7	5.8 ± 0.10 (2)
**10**	347 ± 98	61 ± 22	20 ± 12 (3)
**11**	263 ± 30	50 ± 8.0	19 ± 0.90 (2)

Table S2 *In vitro* selectivity of the PZ and PP series

IC_{50} measured by AlphaScreen after 120 min pre-incubation with compound.

	KDM IC_{50} (μM)								
**Cmpd**	**2A**	**3A**	**4A**	**4B**	**4C**	**5A**	**5B**	**5C**	**5D**	**6B**
**4**	11.3 ± 0.3	8.4 ± 4.1	1.2 ± 0.1	1.1 ± 0.1	1.0 ± 0.3	0.030 ± 0.004	0.020 ± 0.012	0.778 ± 0.037	0.083 ± 0.007	>33
**5**	4.4 ±0.1	2.6 ± 0.1	0.4 ± 0.1	0.7 ± 0.2	0.3± 0.1	0.030± 0.001	0.010 ± 0.005	0.394 ± 0.011	0.048 ± 0.003	0.67 ± 0.01
**6**	>33	>33	2.4 ± 0.1	1.9 ± 0.1	2.3 ± 0.1	0.657 ± 0.007	0.099 ± 0.072	4.570 ± 0.341	0.702 ± 0.047	>100
**7**	>100	5.7 ± 0.4	4.6 ± 0.1	4.1 ± 0.2	1.8 ± 0.1	0.010 ± 0.001	0.007 ± 0.005	0.571 ± 0.034	0.038 ± 0.001	>100
**8**	>33	4.62 ± 0.06	4.70 ± 0.22	9.49 ± 0.05	1.41 ± 0.01	0.052 ± 0.002	0.049 ± 0.001	1.933 ± 0.049	0.189 ± 0.01	9.6 ± 1.7
**9**	>100	17.0 ± 0.4	4.6 ± 0.4	6.3 ± 0.6	3.2 ± 0.1	0.050 ± 0.002	0.065± 0.005	2.192 ± 0.019	0.252 ± 0.01	>100
**10**	>100	9.4± 0.4	12.7 ± 0.6	14.2 ± 1.1	4.0 ± 0.1	0.070 ± 0.008	0.017 ± 0.008	1.273 ± 0.276	0.078 ± 0.007	>100
**11**	>60	9.1 ± 0.8	4.3 ± 0.1	4.6 ± 0.1	1.5 ± 0.1	0.034± 0.002	0.009 ± 0.007	1.038 ± 0.042	0.059 ± 0.002	>100

Table S3. Selectivity Index (SI) of Inhibitors over KDM5B

	SI over KDM5B [a]								
**Compound**	**2A**	**3A**	**4A**	**4B**	**4C**	**5A**	**5C**	**5D**	**6B**
**4**	558	412	58	54	47	1	38	4	>1650
**5**	451	260	41	68	31	3	40	5	67
**6**	>334	>334	24	20	23	7	46	7	>1010
**7**	>14113	806	653	572	253	1	81	5	>14285
**8**	>677	95	97	195	29	1	40	4	196
**9**	>1550	264	71	97	49	1	34	4	>1539
**11**	>6671	1007	479	513	171	4	115	7	>5882
**10**	>5843	547	742	831	234	4	74	5	>11111

[a] Selectivity index calculated dividing IC_{50} of the corresponding compound in the appropriate KDM by the IC_{50} in KDM5B (KDMxx IC_{50}/ KDM5B IC_{50})

Figure S1. Co-crystal structures of compounds 7 and 10 in KDM5A

Superimposition of co-crystal of compound **1** in KDM5A (yellow from PDB ID 6BH4) **in** X-ray co-crystal of compounds **7** (green, PDB ID 6EIY) and **10** (purple, PDB ID 6EJ0) in KDM5B.

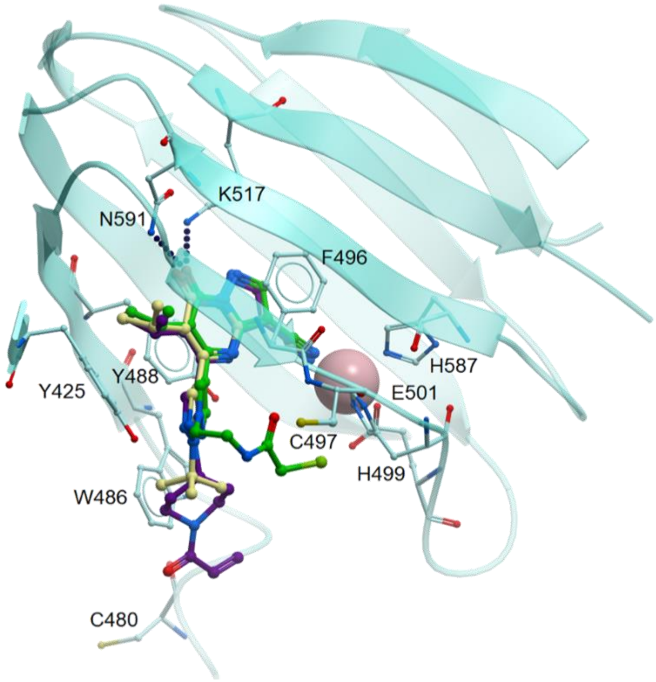

Figure S2. Mass Spectra of compound 6 incubated with KDM5B, KDM4B and KDM5C

A) KDM5B – compound is covalently bound to protein; B) KDM4B – no covalent binding; C) KDM5C – no covalent binding. Dashed oval shows expected mass for covalent adducts.

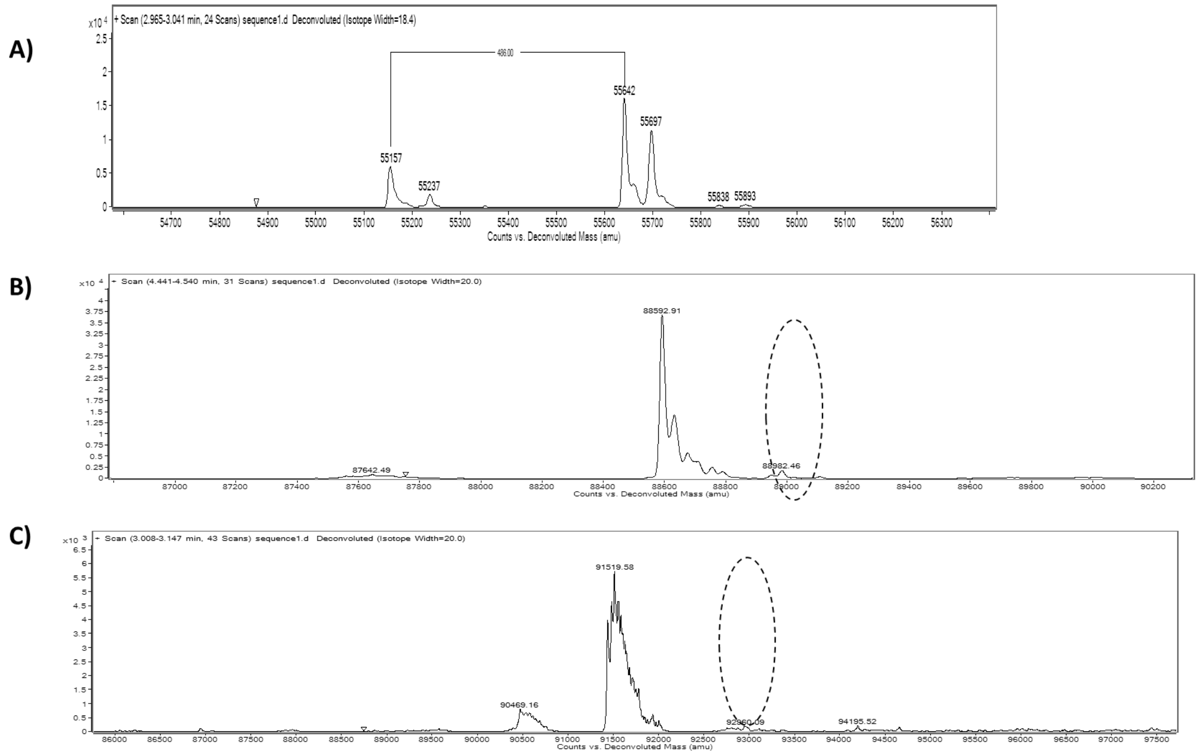

Figure S3. Mass Spectra of compounds incubated with enzymes with known reported covalent inhibitors.

Compounds **4** (red), **7** (orange) and **9** (light blue) incubated at 37 ºC with PCAF (A) and NUDT7 (B). No covalent modifications were observed with either with the PCAF-HAT domain or with NUDT7 for any of the compounds tested after incubation at 37 ºC for 1h. Expected modification peaks for each compounds are indicated with purple arrows.

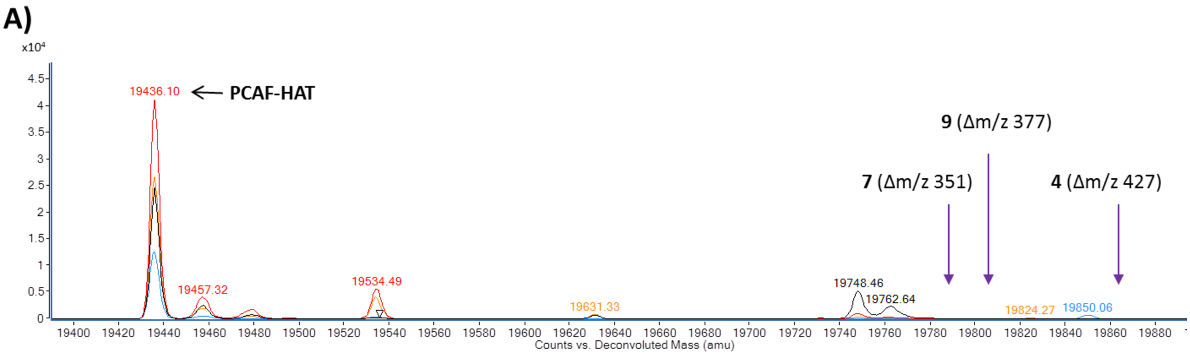

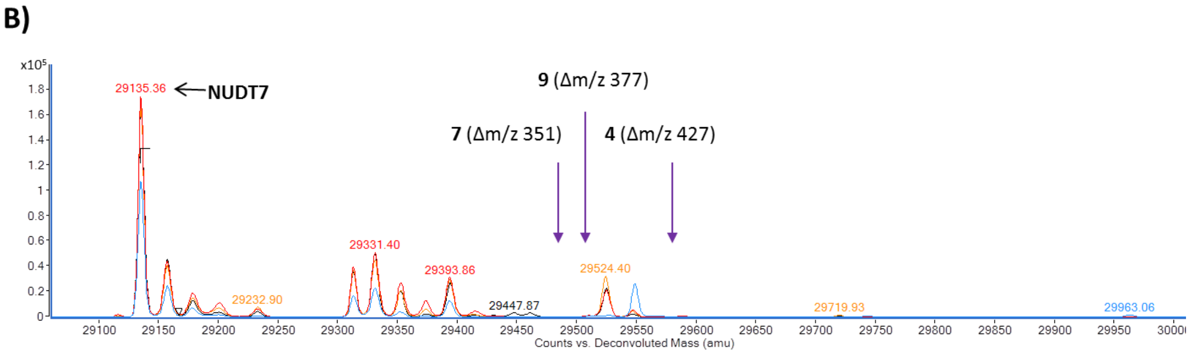

Figure S4. Titration of NanoBRET tracer with KDM5B-NanoLuc

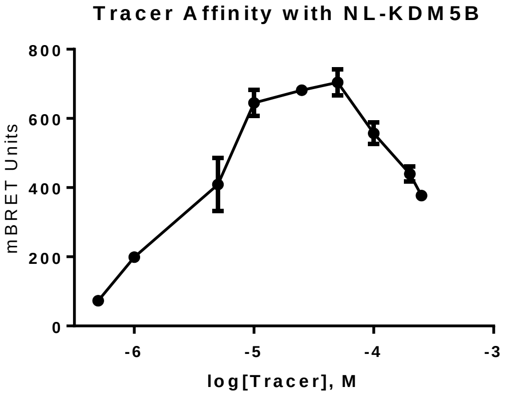

Figure S5. Selectivity of KDM5 NanoBRET tracer

NanoLuc-KDMs with different concentrations of tracer and with or without 20 μM of compound **1.**

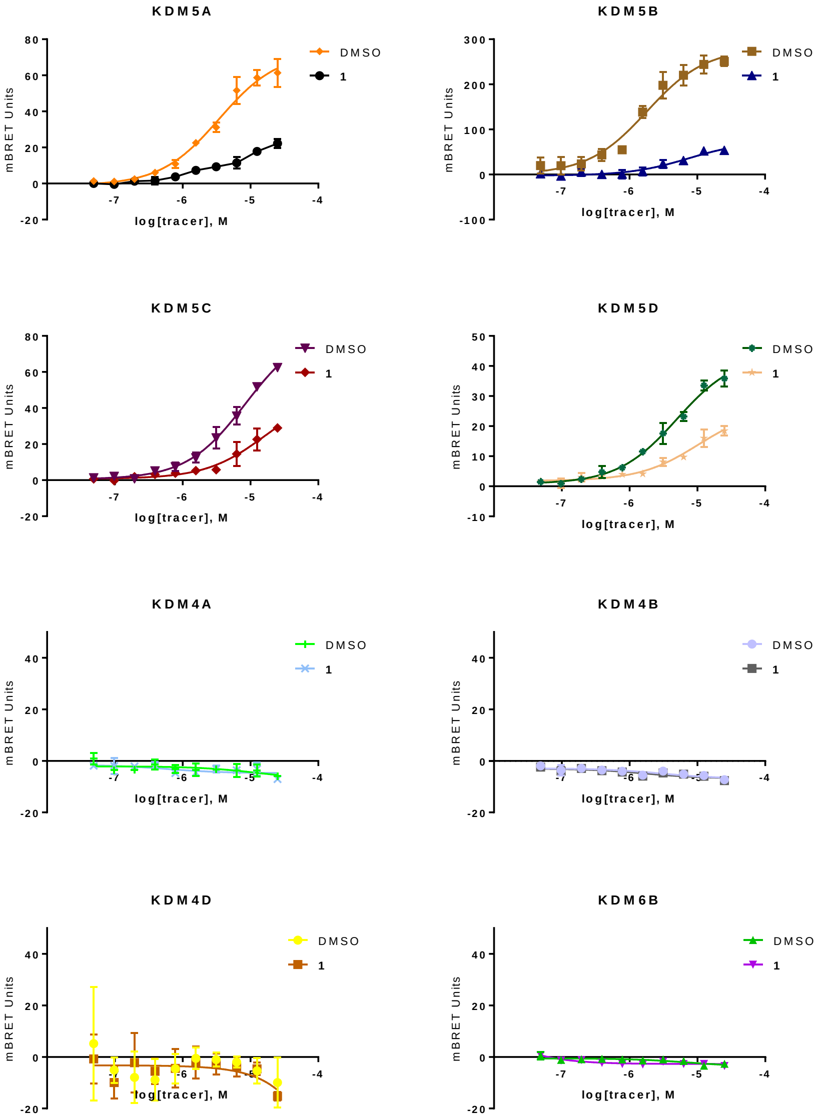

Figure S6. Cellular IC50 in the NanoBRET assay in HEK293 cells for PP and PZ compounds

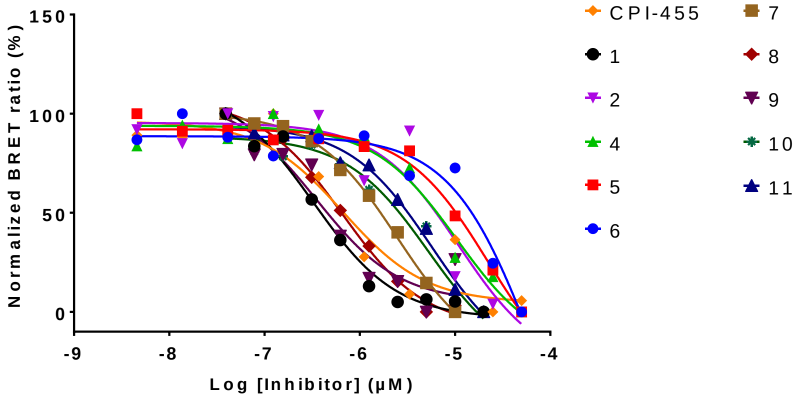

Figure S7. Cytotoxicity of PZ and PP in HEK293 cells

Viability measured by CTG at 72 hrs with compounds and crizontinib as positive control.

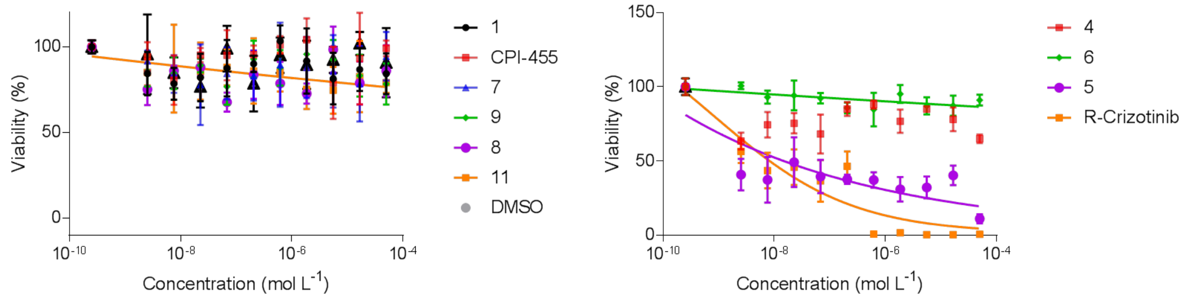

Figure S8 Genomic snapshot of ARID3B

Densities of ChIP-seq reads for H3K4me3 and input in HEK293 cells treated with DMSO and compounds **4**, **7** and **9**.

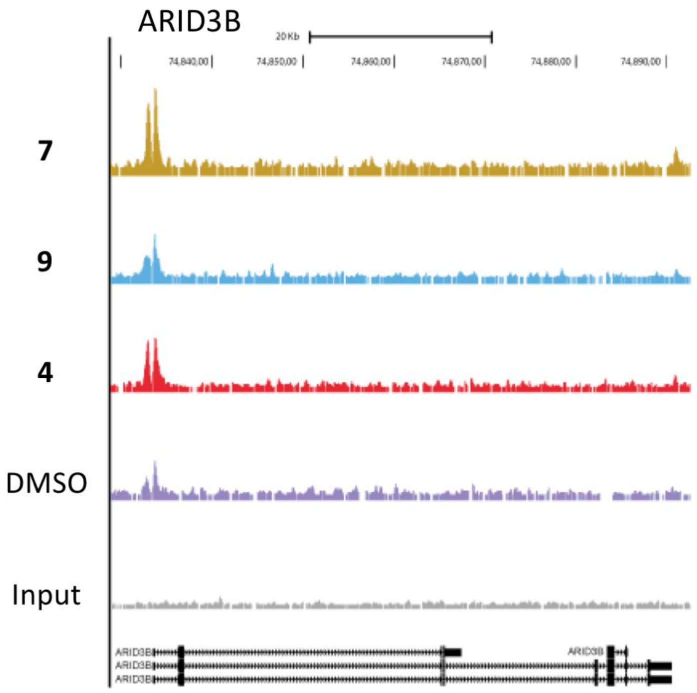

Table S4. AlphaScreen assay conditions for the KDM selectivity panel

Assays were performed as preciously described[5a, 5b] at a 2-OG concentration near the 2-OG Km values determined experimentally.[5c]

Enzyme	[E] nM	[2-OG] µM	[Fe (II)] µM	LAA (µM)	FAS (µM)	[peptide] nM	Peptide substrate	Incubation (min)	Antibody	Source
**KDM2A**	5	10	1	100	1	100	H3(21-44)K36Me2-Biotin	30	Ab9048	Abcam
**KDM3A**	0.25	5	10	100	10	60	H3(1-21)K9Me2-Biotin	5	Ab8896	Abcam
**KDM4A**	2	10	1	100	1	30	Biotin-H3(1-15)K9Me3	10	Ab1220	Abcam
**KDM4B**	2	10	1	100	1	30	Biotin-H3(1-15)K9Me3	10	Ab1220	Abcam
**KDM4C**	0.5	10	1	100	1	30	Biotin-H3(1-15)K9Me3	10	Ab1220	Abcam
**KDM5A**	4	5	10	100	10	100	H3(1-21)K4Me3-Biotin	25	CST 9725S	Cell Signalling Technology
**KDM5B**	2	5	10	100	10	100	H3(1-21)K4Me3-Biotin	20	CST 9725S	Cell Signalling Technology
**KDM5C**	2	5	10	100	10	100	H3(1-21)K4Me3-Biotin	15	CST 9725S	Cell Signalling Technology
**KDM5D**	2	5	10	100	10	100	H3(1-21)K4Me3-Biotin	15	CST 9725S	Cell Signalling Technology

Table S5. Crystallographic statistics for Compounds 3, 7 and 10

**PDB CODE**	**6EJ0**	**6EIN**	**6EIY**
**Compound**	**10**	**3**	**7**
**Space group**	P6522	P6522	P6522
**Unit Cell dimensions**	a, b= 141.82 Å, c = 151.75 Å<br>α, β= 90 °, γ =120°	a, b = 143.47 Å, c = 153.47 Å<br>α, β= 90 °, γ =120°	a, b= 142.38 Å c = 152.17 Å<br>α, β= 90 °, γ =120°
**X-ray source**	DLS I04	DLS I04	DLS I04
**Wavelength (Å)**	0.97950	0.97950	0.97950
**Resolution range (Å)**	51.81-2.06 (2.13-2.06)[a]	44.91-2.11 (2.18-2.11)[a]	51.98-2.15 (2.22-2.15)[a]
**Completeness (%)**	100 (100)	100 (100)	100 (100)
**Multiplicity**	19.8 (20.0)	19.3 (16.6)	19.6 (19.0)
**I/σ I**	21.6/1.4	10.7/1.2	18.7/1.3
**R_{merge}**	0.094/2.41	0.21/2.588	0.11/2.52
**R_{meas}**	0.099/2.53	0.22/2.75	0.12/2.66
**$CC_{1/2}$**	1.0/0.595	0.998/0.537	0.999/0.586
**Unique reflections**	56074(4074)	54032 (3912)	49900 (4901)
**Refinement**			
***R*work/ *R*free (%)**	19.03//21.04	19.63/22.46	18.61/21.54
**RMS (bonds)**	0.008	0.009	0.009
**RMS (angles)**	0.95	0.9	1.02
**B-factors (Å^2)**			
**Protein**	56.56	45.73	55.61
**Ligand/ion**	67.26	52.52	55.60
**Water**	58.18	53.70	56.44
**Ramachandran plot**			
**Favored**	97.32	99.07	96.89
**Allowed**	2.68	0.93	2.89
**Outliers**	0.00	0.00	0.26

[a] Values in the parentheses refer to the outer resolution shell

Figure S9. Electron density of compounds 3, 7 and 10 in co-crystal structures with KDM5B

Blue mesh from 2F0−Fc map at 1.0 sigma.

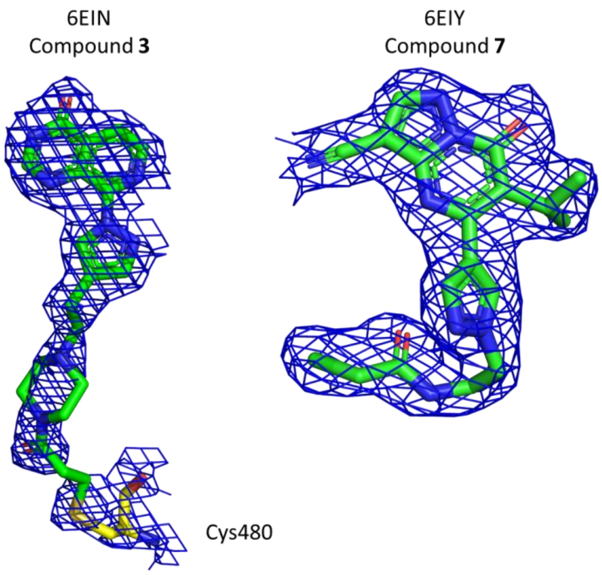

References

[1] V. Bavetsias, R. M. Lanigan, G. F. Ruda, B. Atrash, M. G. McLaughlin, A. Tumber, N. Y. Mok, Y. V. Le Bihan, S. Dempster, K. J. Boxall, F. Jeganathan, S. B. Hatch, P. Savitsky, S. Velupillai, T. Krojer, K. S. England, J. Sejberg, C. Thai, A. Donovan, A. Pal, G. Scozzafava, J. M. Bennett, A. Kawamura, C. Johansson, A. Szykowska, C. Gileadi, N. A. Burgess-Brown, F. von Delft, U. Oppermann, Z. Walters, J. Shipley, F. I. Raynaud, S. M. Westaway, R. K. Prinjha, O. Fedorov, R. Burke, C. J. Schofield, I. M. Westwood, C. Bountra, S. Muller, R. L. van Montfort, P. E. Brennan, J. Blagg, *J. Med. Chem.* **2016**, *59*, 1388-1409.

[2] B. K. Albrecht, S. F. Bellon, V. S. Gehling, J.-C. Harmange, T. Lai, J. Liang, P. Dragovich, D. Ortwine, S. abadie, B. Zhang, J. Kiefer, Genentech, Inc., USA; Constellation Pharmaceuticals, Inc., **2014**.

[3] C. Johansson, S. Velupillai, A. Tumber, A. Szykowska, E. S. Hookway, R. P. Nowak, C. Strain-Damerell, C. Gileadi, M. Philpott, N. Burgess-Brown, N. Wu, J. Kopec, A. Nuzzi, H. Steuber, U. Egner, V. Badock, S. Munro, N. B. LaThangue, S. Westaway, J. Brown, N. Athanasou, R. Prinjha, P. E. Brennan, U. Oppermann, *Nat. Chem. Biol.* **2016**, *12*, 539-545.

[4] A. Kawamura, A. Tumber, N. R. Rose, O. N. King, M. Daniel, U. Oppermann, T. D. Heightman, C. Schofield, *Anal. Biochem.* **2010**, *404*, 86-93.

[5] a) N. R. Rose, E. C. Y. Woon, A. Tumber, L. J. Walport, R. Chowdhury, X. S. Li, O. N. F. King, C. Lejeune, S. S. Ng, T. Krojer, M. C. Chan, A. M. Rydzik, R. J. Hopkinson, K. H. Che, M. Daniel, C. Strain-Damerell, C. Gileadi, G. Kochan, I. K. H. Leung, J. Dunford, K. K. Yeoh, P. J. Ratcliffe, N. Burgess-Brown, F. von Delft, S. Muller, B. Marsden, P. E. Brennan, M. A. McDonough, U. Oppermann, R. J. Klose, C. J. Schofield, A. Kawamura, *J. Med. Chem.* **2012**, *55*, 6639-6643; b) L. Hillringhaus, W. W. Yue, N. R. Rose, S. S. Ng, C. Gileadi, C. Loenarz, S. H. Bello, J. E. Bray, C. J. Schofield, U. Oppermann, *J. Biol. Chem.* **2011**, *286*, 41616-41625; c) R. J. Hopkinson, A. Tumber, C. Yapp, R. Chowdhury, W. Aik, K. H. Che, X. S. Li, J. B. L. Kristensen, O. N. F. King, M. C. Chan, K. K. Yeoh, H. Choi, L. J. Walport, C. C. Thinnes, J. T. Bush, C. Lejeune, A. M. Rydzik, N. R. Rose, E. A. Bagg, M. A. McDonough, T. J. Krojer, W. W. Yue, S. S. Ng, L. Olsen, P. E. Brennan, U. Oppermann, S. Muller, R. J. Klose, P. J. Ratcliffe, C. J. Schofield, A. Kawamura, *Chem. Sci.* **2013**, *4*, 3110-3117.

[6] B. F. Krippendorff, R. Neuhaus, P. Lienau, A. Reichel, W. Huisinga, *J. Biomol. Screen.* **2009**, *14*, 913-923.

[7] a) D. Sims, N. E. Ilott, S. N. Sansom, I. M. Sudbery, J. S. Johnson, K. A. Fawcett, A. J. Berlanga-Taylor, S. Luna-Valero, C. P. Ponting, A. Heger, *Bioinformatics* **2014**, *30*, 1290-1291; b)

Complete References from Manuscript

[7] a) J. Liang, S. Labadie, B. Zhang, D. F. Ortwine, S. Patel, M. Vinogradova, J. R. Kiefer, T. Mauer, V. S. Gehling, J.-C. Harmange, R. Cummings, T. Lai, J. Liao, X. Zheng, Y. Liu, A. Gustafson, E. Van der Porten, W. Mao, B. M. Liederer, G. Deshmukh, L. An, Y. Ran, M. Classon, P. Trojer, P. S. Dragovich, L. Murray, Bioorg. Med. Chem. Lett. 2017, 27, 2974-2981; [13] C. Johansson, S. Velupillai, A. Tumber, A. Szykowska, E. S. Hookway, R. P. Nowak, C. Strain-Damerell, C. Gileadi, M. Philpott, N. Burgess-Brown, N. Wu, J. Kopec,

A. Nuzzi, H. Steuber, U. Egner, V. Badock, S. Munro, N. B. LaThangue, S. Westaway, J. Brown, N. Athanasou, R. Prinjha, P. E. Brennan, U. Oppermann, Nat. Chem. Biol. 2016, 12, 78-80.

[11] M. Vinogradova, V. S. Gehling, A. Gustafson, S. Arora, C. A. Tindell, C. Wilson, K. E. Williamson, G. D. Guler, P. Gangurde, W. Manieri, J. Busby, E. M. Flynn, F. Lan, H. J. Kim, S. Odate, A. G. Cochran, Y. Liu, M. Wongchenko, Y. Yang, T. K. Cheung, T. M. Maile, T. Lau, M. Costa, G. V. Hegde, E. Jackson, R. Pitti, D. Arnott, C. Bailey, S. Bellon, R. T. Cummings, B. K. Albrecht, J. C. Harmange, J. R. Kiefer, P. Trojer, M. Classon, Nat. Chem. Biol. 2016, 12, 531-538.

[12] J. Liang, B. Zhang, S. Labadie, D. F. Ortwine, M. Vinogradova, J. R. Kiefer, V. S. Gehling, J. C. Harmange, R. Cummings, T. Lai, J. Liao, X. Zheng, Y. Liu, A. Gustafson, E. Van der Porten, W. Mao, B. M. Liederer, G. Deshmukh, M. Classon, P. Trojer, P. S. Dragovich, L. Murray, Bioorg. Med. Chem. Lett. 2016, 26, 4036-4041.

[13] V. Bavetsias, R. M. Lanigan, G. F. Ruda, B. Atrash, M. G. McLaughlin, A. Tumber, N. Y. Mok, Y. V. Le Bihan, S. Dempster, K. J. Boxall, F. Jeganathan, S. B. Hatch, P. Savitsky, S. Velupillai, T. Krojer, K. S. England, J. Sejberg, C. Thai, A. Donovan, A. Pal, G. Scozzafava, J. M. Bennett, A. Kawamura, C. Johansson, A. Szykowska, C. Gileadi, N. A. Burgess-Brown, F. von Delft, U. Oppermann, Z. Walters, J. Shipley, F. I. Raynaud, S. M. Westaway, R. K. Prinjha, O. Fedorov, R. Burke, C. J. Schofield, I. M. Westwood, C. Bountra, S. Muller, R. L. van Montfort, P. E. Brennan, J. Blagg, J. Med. Chem. 2016, 59, 1388-1409.

[17] a) T. Machleidt, C. C. Woodroofe, M. K. Schwinn, J. Mendez, M. B. Robers, K. Zimmerman, P. Otto, D. L. Daniels, T. A. Kirkland, K. V. Wood, ACS Chem. Biol. 2015, 10, 1797-1804; b) M. B. Robers, M. L. Dart, C. C. Woodroofe, C. A. Zimprich, T. A. Kirkland, T. Machleidt, K. R. Kupcho, S. Levin, J. R. Hartnett, K. Zimmerman, A. L. Niles, R. F. Ohana, D. L. Daniels, M. Slater, M. G. Wood, M. Cong, Y. Q. Cheng, K. V. Wood, Nat Commun 2015, 6, 10091.

[18] S. B. Hatch, C. Yapp, R. C. Montenegro, P. Savitsky, V. Gamble, A. Tumber, G. F. Ruda, V. Bavetsias, O. Fedorov, B. Atrash, F. Raynaud, R. Lanigan, L. Carmichael, K. Tomlin, R. Burke, S. M. Westaway, J. A. Brown, R. K. Prinjha, E. D. Martinez, U. Oppermann, C. J. Schofield, C. Bountra, A. Kawamura, J. Blagg, P. E. Brennan, O. Rossanese, S. Muller, Epigenetics Chromatin 2017, 10, 9.
